# Human exposure to green spaces and monoterpenes: discovery and use of α-pinene metabolites as biomarkers

**DOI:** 10.1016/j.envint.2025.109710

**Published:** 2025-08-04

**Authors:** Zhengzhi Xie, Saurin R. Sutaria, Jin Y. Chen, Hong Gao, Daniel J. Conklin, Rachel J. Keith, Sanjay Srivastava, Pawel Lorkiewicz, Aruni Bhatnagar

**Affiliations:** a Christina Lee Brown Envirome Institute, University of Louisville, USA; b Superfund Research Center, University of Louisville, USA; c American Heart Association-Tobacco Regulation and Addiction Center, University of Louisville, USA; d Department of Chemistry, Bellarmine University, USA; e Division of Environmental Medicine, Department of Medicine, University of Louisville, Louisville, KY 40202, USA; f Department of Chemistry, University of Louisville, USA

**Keywords:** Greenness exposure, Monoterpene, biogenic volatile organic compounds (bVOC), α-pinene, LC-MS, Data-independent acquisition (DIA), Integrated library-guided analysis (ILGA)

## Abstract

Exposure to green spaces has been linked to numerous health benefits, including a reduction in the risk of cardiovascular disease and lower mortality rates. However, to better understand how green spaces influence human health, it is essential to have valid, quantitative measures of greenness exposure. Building on our previous identification of urinary limonene metabolites as potential biomarkers of exposure, we now investigate α-pinene, another abundant plant-emitted monoterpene, to identify and quantify its urinary metabolites and evaluate their suitability as biomarkers. We used liquid chromatography-high resolution mass spectrometry (LC-HRMS) to analyze samples of human urine collected following either controlled α-pinene inhalation or real-world greenness exposure. Through a combination of pseudo-targeted and untargeted analyses, we discovered 22 α-pinene metabolites post-inhalation including nine novel structures, with two confirmed against synthetic standards. Relative quantitation of the urinary levels of the metabolites was used to estimate their kinetic parameters. A 4 h exposure to a forest environment resulted in significant increases in the most abundant metabolites. This suggests that α-pinene metabolites, specifically myrtenic acid glucuronide and dihydromyrtenic acid glucuronide, may serve as valid biomarkers when assessing individual exposure to green environments. When combined with other subjective and objective measures, these novel urinary biomarkers promote a more comprehensive assessment of exposure to greenness.

## 1. Introduction

Green spaces, specifically forested environments, are widely recognized for their health benefits (Nguyen et al., 2021; WHO, 2016). Living in areas of high surrounding greenness has been associated with improved cardiovascular health (Yeager et al., 2020), enhanced immune responses (Andersen et al., 2021), and lower mortality rates (James et al., 2016). Even brief exposure to greenness can improve physical and mental well-being (Keith et al., 2024). However, despite increasing recognition of the health benefits of greenness, there are few, if any, robust and quantitative biomarkers of exposure to natural greenness and vegetation. (Hart et al., 2022). Forested areas and environments with abundant trees are particularly high in biogenic volatile organic compounds (bVOCs), especially monoterpenes (Yang et al., 2021). To address this in our previous research, we identified 18 urinary metabolites of limonene, a common plant-derived bVOC, following a controlled inhalation experiment (Xie et al., 2024). Three of the most abundant metabolites of limonene showed elevated concentrations after exposure to forested area, demonstrating the use of these metabolites as biomarkers for assessing greenness exposure (Xie et al., 2024).

**Fig. 1.**
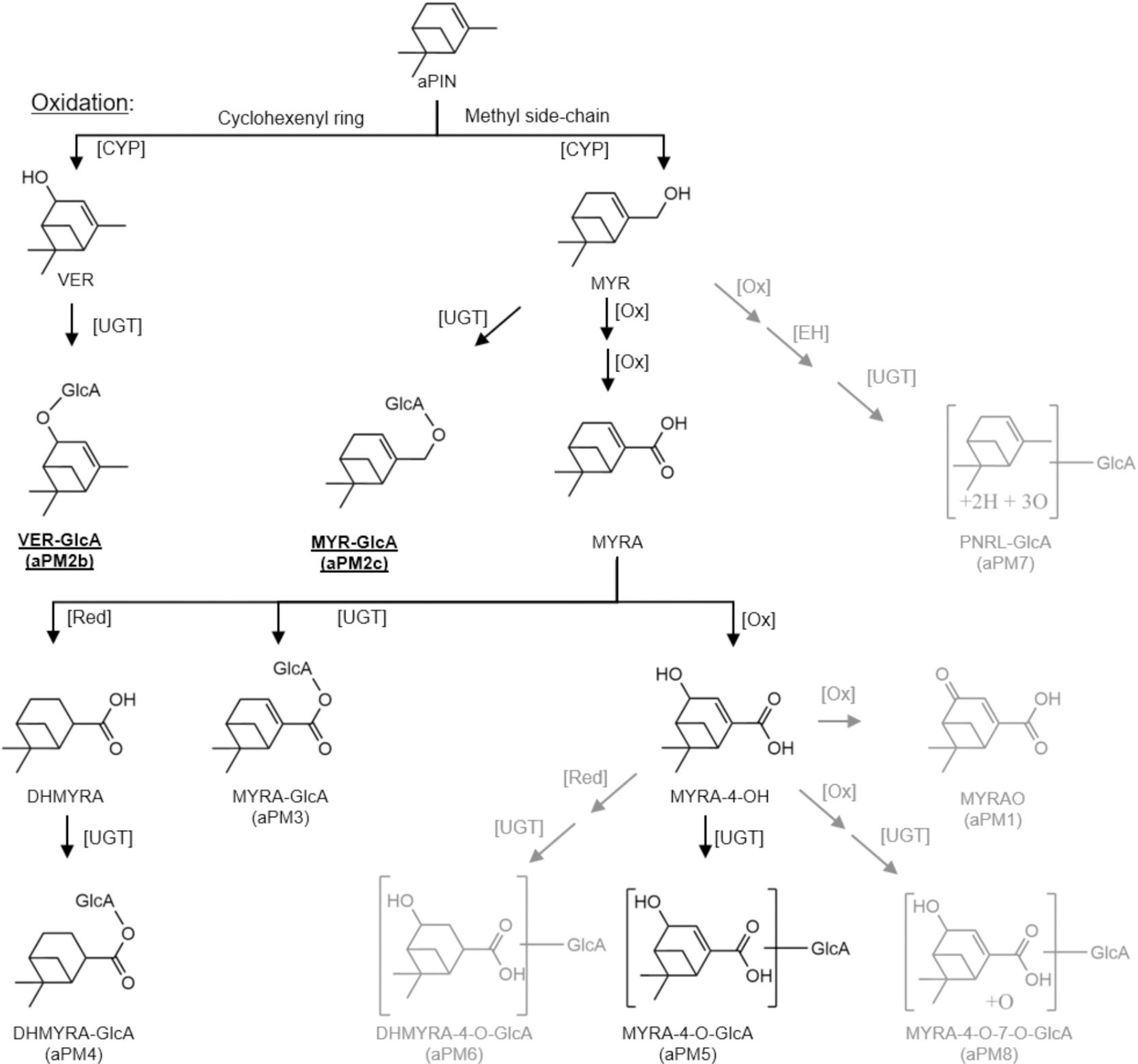
α-Pinene metabolism pathway in human. Only detected metabolites (with aPM numbers, see Table 1) and their intermediate products are shown in the figure. The structures in black are metabolites previously reported in human urine or their predicted glucuronides. The structure in gray are metabolites detected via untargeted analysis. Structures of aPM7 and aPM8 were tentatively assigned, and others were assigned. While the structures of aPM2b and aPM2c have been confirmed through comparison with synthesized standards, further experimental evidence is needed to confirm other structures, especially those detected via untargeted analysis. Abbreviations: Glucuronic acid (GlcA); epoxide hydrolase (EH); oxidation (Ox); reduction (Red); UDP-glucuronosyl transferase (UGT); cytochromes P450 (CYP); The abbreviations for names of the metabolites can be found at Tables S3 and S4.

Nevertheless, exposure assessment of other biogenic VOCs remains difficult as little is known about their metabolites and how they could be identified, measured, and related to greenness exposure. Therefore, we examined the metabolism of α-pinene, which is a major bVOC emitted by both coniferous and deciduous trees (Borsdorf et al., 2023), and accounts for more than half of the global monoterpene emissions, (Bagchi et al., 2020). This bicyclic monoterpene shares its molecular formula (C_10_H_16_) with limonene, a monocyclic monoterpene, but differs significantly in structure. While α-pinene comprises two rings and a single carbon–carbon double bond within a six-membered ring (Fig. 1), limonene’s structure includes single ring and two double bonds, with one double bond on the sidechain, providing a primary site of oxidative metabolism in humans (Fig. S1). These structural differences drive metabolic divergence between the two monoterpenes (Schmidt and Göen 2017a; b). Characterizing α-pinene metabolites (aPM) offers a novel means to capture exposures to greenness. Additionally, identification of α-pinene metabolites is important because emerging evidence suggests that some of its metabolites may possess bioactive properties (Jin et al., 2023). Thus, identification of the metabolic transformation of α-pinene may shed new light on the structural-activity relationships of the monoterpenes and inform future drug-discovery investigations.

Phase I metabolism of α-pinene has been studied before and several metabolites have been identified (de Alvarenga et al., 2023; Eriksson and Levin 1996; Ishida et al., 1981; Li et al., 2013; Schmidt et al., 2013; Schmidt and Göen 2017a; Southwell et al., 1980; Waidyanatha et al., 2022; Yang et al., 2023) (Fig. 1). However, most of these metabolites were identified post-enzymatic hydrolysis (Schmidt et al., 2013; Schmidt and Göen 2017a), which removes phase II conjugation with glucuronic acid, sulfate, or other endogenous molecules. Therefore, phase II metabolites have not been directly identified, characterized, or measured. Our investigations aimed to bridge this gap by systematically identifying and quantifying individual phase II metabolites and assessing their utility as biomarkers for greenness exposure. Building on this foundation, controlled single-monoterpene exposures were combined with real-world greenness exposure to generate mechanistic, time-resolved data on α-pinene metabolism and kinetics, and to apply these insights in an environmentally relevant context. In addition, to strengthen confidence in biomarker selection and biological interpretation, a controlled mouse study was conducted to verify key urinary metabolites and clarify enantiomer specificity under defined exposure conditions. Together, these experiments support the use of α-pinene metabolites as short-term exposure markers and provide a framework for broader investigations across monoterpenes.

## 2. Materials and methods

### 2.1. Chemicals and reagents

(−)-α-pinene (CAS# 7785-26-4) and (+)-α-pinene (CAS# 7785-70-8) for murine exposures were acquired from Sigma-Aldrich, Inc. (St. Louis, MO). (±)-α-pinene (CAS# 80-56-8) for human exposure was obtained from True Terpenes (Hillsboro, OR). Acetonitrile, water (both UHPLC-MS grade), formic acid (LC-MS grade), and Infinity^™^ Creatinine Liquid Stable Reagent were purchased from Thermo Fisher Scientific Inc., Waltham MA. For synthesis of verbenol glucuronide (VER-GlcA) and myrtenol glucuronide (MYR-GlcA), (S)-*cis*-verbenol (95 %) and (−)-myrtenol (95 %) were purchased from Sigma-Aldrich (St. Louis, MO). Human UGT1A9 Supersomes, UGT Reaction Mix Solution A (25 mM uridine 5′-diphospho-glucuronic acid, UDPGA), and UGT Reaction Mix Solution B (40 mM MgCl_2_ + 0.125 mg/mL alamethicin) were obtained from Discovery Life Sciences (Woburn, MA).

### 2.2. Human urine sample collection

Human urine samples were collected using methods reported previously (Xie et al., 2024). Briefly, for controlled inhalation experiments, eight healthy volunteers were exposed to α-pinene in a custom-built exposure chamber via inhalation from a 15 ml conical tube, containing approximately 100 μl α-pinene, for a duration of 10 min. Urine samples were collected before exposure (0 h) and 0.5, 1, 2, 3, and 4 h after exposure. For greenness exposure, participants spent 4 h in Charlestown State Park, IN, USA, an area characterized by deciduous trees, including sugar maple, red maple, black cherry, and white ash (Lynch 2016; Maxwell and Thomas 2003). Urine samples were collected both before (0 h) or 4 h after exposure. After collection, the urine samples were centrifuged and stored at −80 °C before analysis. These studies were approved by the University of Louisville Institutional Review Board (IRB 22.0543 and IRB 16.0944), and consent was obtained from participants prior to any procedures. Demographic information, including sex, age and race, can be found in Table S1.

### 2.3. LC-MS analysis of urine samples

The UPLC-QTOF-MS DIA analysis of urine samples followed the procedure reported previously (Xie et al., 2024). Briefly, after 1:10 dilution, urine samples were separated using an Acquity I-Class UPLC system with an Acquity Premier CSH C18 UPLC column (Waters, MA) with a gradient comprising 0.1 % formic acid in water as the solvent A and 0.1 % formic acid in acetonitrile as the solvent B. Mass Spectrometry Elevated Energy (MS^E^) data were collected using a Waters Synapt XS MS (Waters, MA) with an ESI ion source operated in the negative mode. Subsequent analyses of raw files were performed using UNIFI 1.9 and Progenesis QI 3.0 (both from Waters, MA). To validate LC-MS performance, one quality control (QC) sample, prepared by pooling equal amount of each urine sample, was injected after every ten urine sample injections. Table S2 provides details of retention time shifts (ΔRt) and mass errors (Δ*m*/*z*) observed for aPM in QC samples.

For structural confirmation of phase II aPM, LC-MS/MS data were collected from human urine following α-pinene inhalation under conditions similar to the UPLC-QTOF-MS DIA analysis. The resulting MS/MS spectra were averaged around their respective Rt and examined for characteristic peaks associated with glucuronides and their aglycones.

### 2.4. Selection of candidate aPM peaks

Two complementary approaches were used to select candidate aPM peaks from LC-MS data acquired from urine after α-pinene exposure: an Integrated Library-Guided Analysis (ILGA) pseudo-targeted workflow and an untargeted method (Fig. S2).

The ILGA workflow (Xie et al., 2023; Xie et al., 2024) utilized a scouting library containing structures of known and proposed aPM. To compile aPM, we conducted a systematic search with the SciFinder^n^ database for three CAS numbers: (1) 80-56-8 for (±)-α-pinene, (2) 7785-70-8 for (+)-α-pinene, and (3) 7785-26-4 for (−)-α-pinene. Molecular structure files (.mol) for identified metabolites were downloaded and imported into a custom-built library within the UNIFI software. To expand the library, we incorporated both known aPM and newly proposed phase II metabolites based on established biotransformation pathways (Table S3). Following prior reports (Rinaldi de Alvarenga et al., 2022; Xie et al., 2024), we included glucuronidation, sulfation, glycine conjugation, and taurine conjugation products for acidic phase I metabolites, and glucuronidation and sulfation products for alcoholic phase I metabolites. Methylation and acetylation products were excluded, as they are unlikely to be detected by ESI-MS in negative mode. The finalized library, containing structural predictions and expected *m*/*z* values, was applied in ILGA data processing using the UNIFI software for candidate peak selection and metabolite identification.

Complimentary to the ILGA workflow, an untargeted approach was used to process raw LC-MS results following procedures reported previously (Zhang et al., 2016). After peak alignment, LC-MS peak picking, and deconvolution, the output from both analyses was exported into similar flat (.csv) files containing LC-MS features (*m*/*z* and Rt) along with their responses, and these files were then combined for the candidate metabolite selection process. The selection criteria for a feature to be qualified as an aPM candidate were as follows: 1) The response of candidate peaks must significantly increase following α-pinene inhalation (p < 0.05). This was assessed by normalizing peak responses to urinary creatinine levels and then comparing the normalized values before and after exposure. 2) Peaks with a maximum response below 1000 were excluded from further analysis. 3) Any features identified as in-source fragments, adducts, or ringing peaks were also excluded.

### 2.5. Peak assignment

Each *m*/*z* value was assigned a unique structure unless experimental data indicated the presence of multiple isomers. Solely relying on *m*/*z* matching was insufficient due to structural isomerism. To incorporate both ILGA and untargeted analyses, we revised our peak assignment approach based on prior LC-MS characterization of limonene metabolites (Xie et al., 2024). For peaks detected in untargeted analysis without a direct match in the library, putative structures were proposed based on characteristic mass shifts relative to known metabolites with strong correlation in intensity. A detailed description of these five annotation rules is provided in Supplementary Materials (Fig. S3). Following assignment, metabolite name, formula, and retention time (Rt) were used to analyze urine samples from the greenness exposure experiment. Isomers sharing the same *m*/*z* but differing in Rt were treated as distinct entities.

### 2.6. Mouse exposures to enantiomers of α-pinene

Mouse urine was collected after exposures to either (+) or (−) α-pinene to distinguish between metabolites of different enantiomers of α-pinene. Adult male mice (C57BL/6) were intraperitoneally administered with 50 mg/kg body weight of (+)-α-pinene or (−)-α-pinene dissolved in 100 μl sterile saline with 1 % Tween 80. Post-injection, mice had access to drinking water and food containing 3 % glucose and 0.125 % saccharin, and their urine was collected overnight in graduated cylinders at 4 °C (Lynch et al., 2020). After centrifugation, urine samples were stored at −80 °C until subjected to LC-MS analysis with the same methodology as human urine (Xie et al., 2024). The procedure was approved by the Institutional Animal Care and Use Committee of the University of Louisville (IACUC #22204).

### 2.7. Synthesis of verbenol glucuronide (VER-GlcA) and myrtenol glucuronide (MYR-GlcA)

VER-GlcA and MYR-GlcA were synthesized via enzymatic glucuronidation of their aglycone, verbenol (VER) and myrtenol (MYR). Briefly, individual stock solutions of 100 mM VER and MYR were prepared in methanol. The reaction vial contained 200 μL of 50 mM Tris-HCl buffer (pH 7.5), 4 μL of VER or MYR stock solution, 4 μL of UGT1A9, and 160 μL of UGT Reaction Mix Solution B. The vial was pre-incubated at 37 °Cfor 10 min, then 32 μL of UGT Reaction Mix Solution A was added making the final reaction volume to be 400 μL. The percent organic solvent in the mixture was not more than 1 % in volume. Next, the vial was incubated at 37 °Cfor 24 h, and the reaction was terminated by adding 100 uL of acetonitrile. Afterward, the mixture was centrifuged at 10,000 rpm for 5 min and the supernatant was collected for UPLC-QTOF/MS analysis.

### 2.8. Relative quantification of aPM and statistical analysis

Relative quantification of aPM was performed based on the peak responses normalized to urinary creatinine levels. Urinary creatinine levels were measured using an Ace Axcel Clinical Chemistry System with ACE Creatinine Reagent from Alfa Wassermann (West Caldwell, NJ). Statistical analyses, including ANOVA and Student’s t-tests, were conducted using SAS 9.4 (SAS Institute, NC) to explore differences in aPM across various time points. To address the right-skewed distributions observed in normalized responses, a log transformation was performed to improve normality. A threshold for statistical significance was defined as p < 0.05.

### 2.9. Kinetic modeling

To examine the generation and elimination of each aPM, we employed a nonlinear mixed-effects model (NLME), as detailed in previous work (Xie et al., 2024). The analysis utilized a pseudo one-compartment model derived from established methodologies (Barbeau et al., 2018; Lou et al., 2009; Lutier et al., 2016) to describe the general patterns of metabolite appearance and elimination (Xie et al., 2024):

(1) $$C_{ij}=\frac{Ka_{i}\;\times \;F_{i}\;\times \;D}{V\left(Ka_{i}\;-\;Ke_{i}\right)}\left(e^{-Ke_{i}\times t_{j}}-e^{-Ka_{i}\times t_{j}}\right)+\varepsilon_{ij}$$

In this equation, i represents the participant index, j the urine sample index, C the creatinine-normalized peak response, and t the sample collection time (h).Ka denotes the first-order generation constant ($h^{-1}$), and Ke denotes the first-order elimination rate constant $\left(h^{-1}\right)$. F indicates the fraction of specific aPM relative to absorbed α-pinene. The variables D and V refer to the absorbed α-pinene amount and the distribution volume, respectively. Because input data were derived from relative quantification, absolute values of D, V, and F were not available. For simplicity, D and V were fixed at 100 and 1, respectively, and F was normalized relative to the sum of all detected aPM. The error term ε accounts for residual variation. It was assumed that Ka, Ke, and F were independent and followed a normal distribution across participants (Xie et al., 2024).

## 3. Results

### 3.1. Discovery and identification of phase II aPM

Candidate aPM peaks were selected from the LC-MS results of human urine after α-pinene inhalation (Table 1). These peaks were then assigned structures based on their *m*/*z* and Rt. To achieve comprehensive annotation and assignment, we combined results from pseudo-targeted analysis, which utilized a scouting library with known and deduced metabolites, with untargeted analysis for identifying novel and unexpected metabolites. The structures of abundant metabolites were subsequently validated using LC-MS/MS, and further confirmed by comparison with synthesized aPM (Fig. S2).

#### 3.1.1. Pseudo-targeted analysis

ILGA-based analysis was conducted using a scouting library of 29 aPM, including 10 phase I metabolites (de Alvarenga et al., 2023; Schmidt et al., 2013; Schmidt and Göen 2017a) and 19 phase II conjugates (glucuronides, sulfates, taurine, and glycine conjugates) (Table S3). These metabolic transformations have been previously observed in other monoterpenes, including limonene (Rinaldi de Alvarenga et al., 2022) and menthol (de Alvarenga et al., 2023), supporting their expected occurrence in α-pinene metabolism.

Human urine samples collected after α-pinene inhalation were analyzed by LC-HRMS to discover candidate peaks for aPM (Fig. S2). Querying the scouting library revealed 624 peaks within 20 ppm of the theoretical *m*/*z* for [aPM-H]^−^. Creatinine-normalized responses were tracked across multiple time points, and LC-MS peaks with significant increases after inhalation were selected for further evaluation. Through this process, 17 aPM peaks were discovered using ILGA, with 4 unique *m*/*z* values: 327.1449, 341.1242, 343.1398, and 357.1191, represented by aPM2, aPM3, aPM4, and aPM5 in Table 1.

#### 3.1.2. Untargeted analysis

Untargeted analysis was used to detect unexpected or novel aPM. Among ~20,000 LC-MS features detected after peak alignment and deconvolution, 13 passed all selection criteria (Section 2.4) and were further annotated as aPM. Eight of these were also found in ILGA-based analysis in the previous section, while five were uniquely detected through untargeted analysis. These metabolites corresponded to four unique *m*/*z* values: 179.0714, 359.1348, 361.1504, and 373.1140, represented by aPM1, aPM6, aPM7, and aPM8 in Table 1. More detailed information about these aPM can be found in Table S4.

#### 3.1.3. Peak annotation

In total, 22 aPM were annotated (Table 1). Metabolites with multiple chromatographic peaks but the same *m*/*z* were assigned separate isomeric designations (e.g., aPM2a, aPM2b, aPM2c) based on retention time differences. Among these, myrtenic acid-4-one (aPM1) was the only phase I metabolite, while the remaining 21 were phase II glucuronides. Most glucuronides existed as multiple isomers, including those of aPM2, aPM3, aPM4, and aPM5. In contrast, aPM6 and aPM8 were each associated with a single LC-MS peak, suggesting the presence of only one isomer. Overall, out of 9 annotated structures, seven were assigned and two were tentatively assigned.

#### 3.1.4. Structural confirmation using LC-MS/MS

**Fig. 2.**
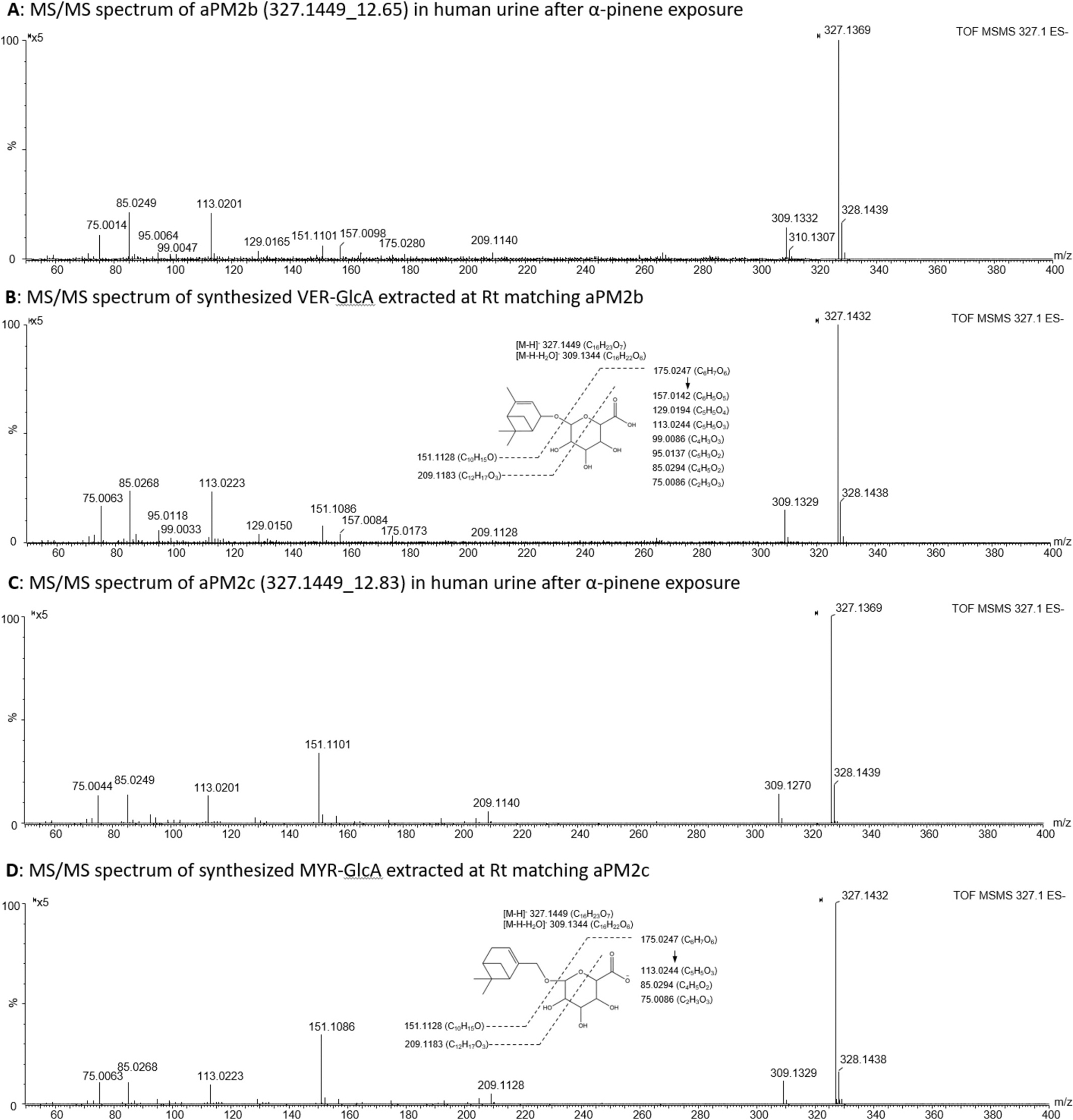
Comparison of MS/MS spectra of α-pinene metabolites (aPM) in human urine and synthesized compounds (**A** and **C** are MS/MS spectra of aPM2b and aPM2c in human urine after α-pinene exposure. **B** and **D** are MS/MS spectra of synthesized VER-GlcA and MYR-GlcA. The inserts are proposed fragmentation patterns of the two synthesized compounds.).

To validate aPM structures, LC-MS/MS data were collected for abundant metabolites (defined by the kinetic analysis at next section), including aPM2b, aPM3b, aPM4d, and aPM4e. Their MS/MS spectra confirmed glucuronide conjugation via peaks representing the glucuronide moiety at 175.0247 and its fragments at 157.0142, 113.0244, 85.0294, and 75.0086 (Evich et al., 2022; Han et al., 2013; Rinaldi de Alvarenga et al., 2022; Scapolla et al., 2012; Xie et al., 2023). Aglycone structures were inferred from reported phase I metabolites (Schmidt and Göen 2017a) and *m*/*z* values corresponding to a neutral loss of 176.0321 (Evich et al., 2022). Based on the scouting library (Table S3), aPM2b was identified as either verbenol glucuronide (VER-GlcA) and myrtenol glucuronide (MYR-GlcA). Synthesized metabolite standards were analyzed alongside post-inhalation urine samples, confirming aPM2b as VER-GlcA (327.1449_12.83) and aPM2c as MYR-GlcA (327.1449_12.83) (Fig. 2). Further structural details are provided in Supplementary Materials.

### 3.2. Relative quantification and kinetics of aPM

**Fig. 3.**
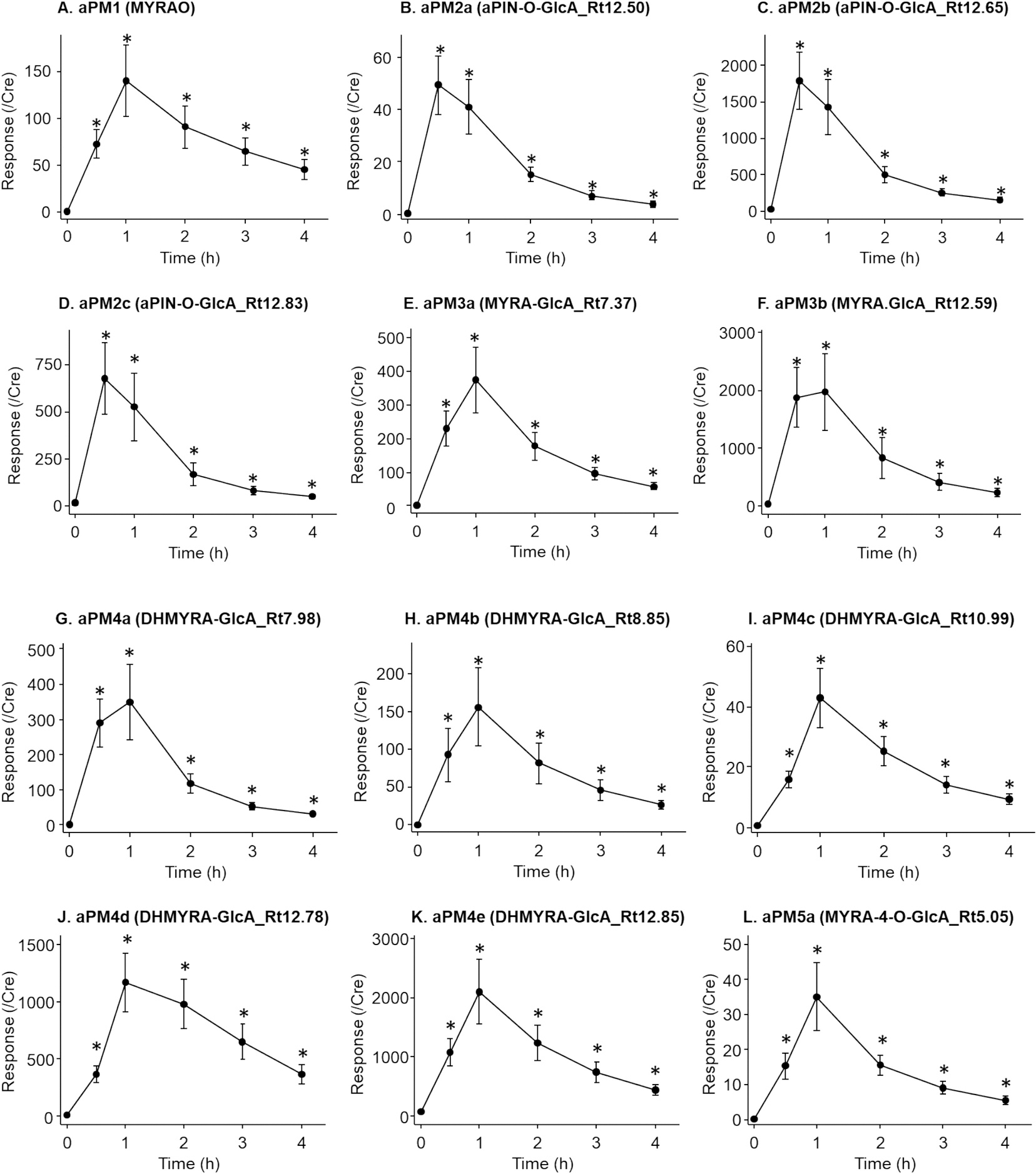

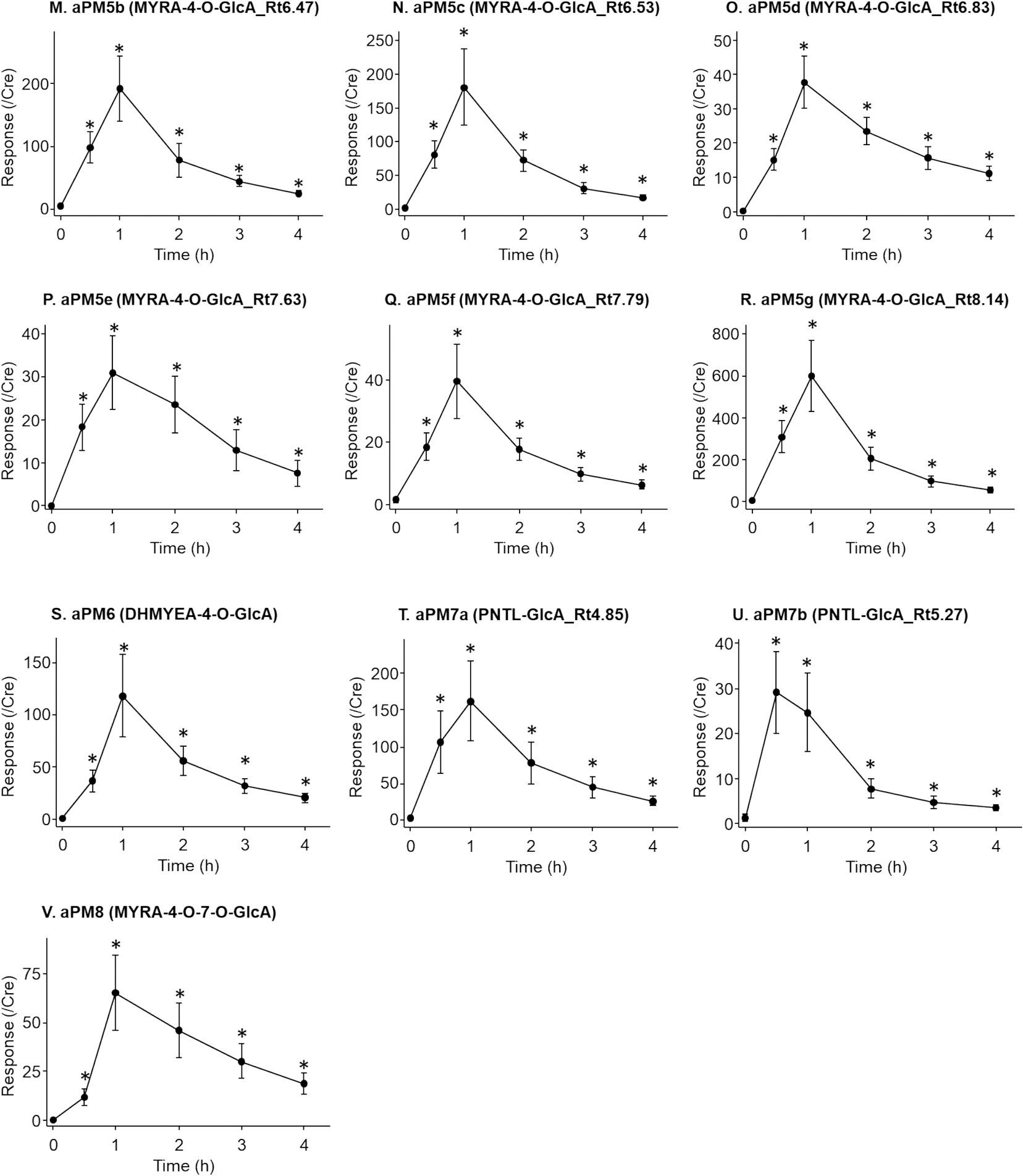
Time courses of 22 aPM in human urine collected after α-pinene inhalation. **A.** aPM1 (MYRAO); **B**. aPM2a (aPIN-O-GlcA_Rt12.50); **C**. aPM2b (aPIN-O-GlcA_Rt12.65); **D**. aPM2c (aPIN-O-GlcA_Rt12.83); **E**. aPM3a (MYRA-GlcA_Rt7.37); **F**. aPM3b (MYRA.GlcA_Rt12.59); **G**. aPM4a (DHMYRA-GlcA_Rt7.98); **H**. aPM4b (DHMYRA-GlcA_Rt8.85); **I**. aPM4c (DHMYRA-GlcA_Rt10.99); **J**. aPM4d (DHMYRA-GlcA_Rt12.78); **K**. aPM4e (DHMYRA-GlcA_Rt12.85); **L**. aPM5a (MYRA-4-O-GlcA_Rt5.05); **M**. aPM5b (MYRA-4-O-GlcA_Rt6.47); **N**. aPM5c (MYRA-4-O-GlcA_Rt6.53); **O**. aPM5d (MYRA-4-O-GlcA_Rt6.83); **P**. aPM5e (MYRA-4-O-GlcA_Rt7.63); **Q**. aPM5f (MYRA-4-O-GlcA_Rt7.79); **R**. aPM5g (MYRA-4-O-GlcA_Rt8.14); **S**. aPM6 (DHMYEA-4-O-GlcA); **T**. aPM7a (PNTL-GlcA_Rt4.85); **U**. aPM7b (PNTL-GlcA_Rt5.27); **V**. aPM8 (MYRA-4-O-7-O-GlcA). Human urine samples were collected before and after α-pinene inhalation, and analyzed using UPLC-QTOF-MS. For each aPM, peak responses were normalized to urinary creatinine levels and plotted. Filled circles and error bars are mean and SE respectively. Student’s *t*-test was performed after log-transformation. (*: p < 0.05).

Time courses of urinary levels for the 22 aPM, quantified as peak intensity normalized to creatinine, are shown in Fig. 3. Most aPM peaked at 1 h post-inhalation and declined gradually. Notably, three aPM2 isomers peaked earlier at 0.5 h, reflecting upstream positions in the metabolic pathway of their aglycones (Fig. 1). Peak intensities ranged from under 4,000 for aPM5e to over 400,000 for aPM2b (Table S5). The four most abundant aPM— aPM2b, aPM3b, aPM4d, and aPM4e—comprised 73.4 % of the total response, each contributing more than 10 % (Table S5). These findings align with prior reports identifying their aglycones, including myrtenol (MYR), verbenol (VER), myrtenic acid (MYRA), and dihydromyrtenic acid (DHMYRA), as major urinary aPM post enzymatic hydrolysis (Schmidt and Göen 2017a).

The results of kinetic analysis, summarized in Table 2, align closely with the relative quantification data. Curves representing the best fit for aPM3b, the most prevalent aPM, are shown in Fig. S4. The fraction of each metabolite generated from the total absorbed α-pinene dose (F) corresponded to its peak response proportion (Fig. S5A). The four most abundant aPM — aPM2b, aPM3b, aPM4d, and aPM4e — exhibited the highest F values (>10 %) and collectively accounted for 71 % of the total F, confirming their status as major urinary metabolites and promising biomarker candidates. Plotting generation rate (Ka) against elimination rate (Ke) revealed two groups: one with high Ka and Ke (Ka > 2.5, Ke > 1.15), including fast-metabolized compounds like aPM2 isomers and aPM7b (peak time 0.5 h), and another with slower rates, peaking at 1 h (Fig. S5B). Elimination half-times (t_1/2_) ranged from 0.50 h for fast metabolized compound, like aPM2c, to 1.51 h for slow metabolized compound, like aPM4c (Table 2).

### 3.3. Metabolism of α-pinene enantiomers in mice

Among the 22 human aPM, 16 were also observed in mouse urine after exposure to either (1S)-(+) or (1R)-(−) isomers of α-pinene, indicating substantial overlap in metabolic pathways between the species (Table S6). The four most abundant human metabolites — aPM2b, aPM3b, aPM4d, and aPM4e — were all present in murine samples. Six human aPM present were absent in murine samples. All of them were minor metabolites with human *F* values below 2 %, except aPM5g (F ~ 5.1 %). Interestingly, five of these six undetected aPM contain the MYRA-4-O moiety, suggesting a species-specific metabolic difference. Unlike humans, mice appear to be unable to oxidize MYRA to MYRA-4-OH (Fig. 1).

Enantiomer specificity was observed in some aPM (Table S6) and apparently related to specific branches of the metabolic pathways (Fig. 1). Metabolites containing the HMYRA moiety (aPM4 and aPM6) were predominantly associated with the (1S)-(+) isomer, with R/(R + S) values ranging from 0–24 %. In contrast, aPM with the Pinanetriol (PNRL) moiety (aPM7) were more abundant following exposure to the (1R)-(−) isomer, with R/(R + S) values between 85–100 %. Seven aPM (aPM3a, aPM4a–d, aPM5a, aPM5e) were present only after exposure to the (1R)-(−) isomer, each with R/(R + S) at 0 %. Conversely, aPM5d and aPM7b were exclusively detected post-(1S)-(+) exposure, with R/(R + S) values at 100 %. aPM containing the aPIN-O moiety (aPM2) showed no significant enantiomer bias (R/(R + S): 42–60 %).

### 3.4. aPM levels in human urine after exposure to greenness

**Fig. 4.**
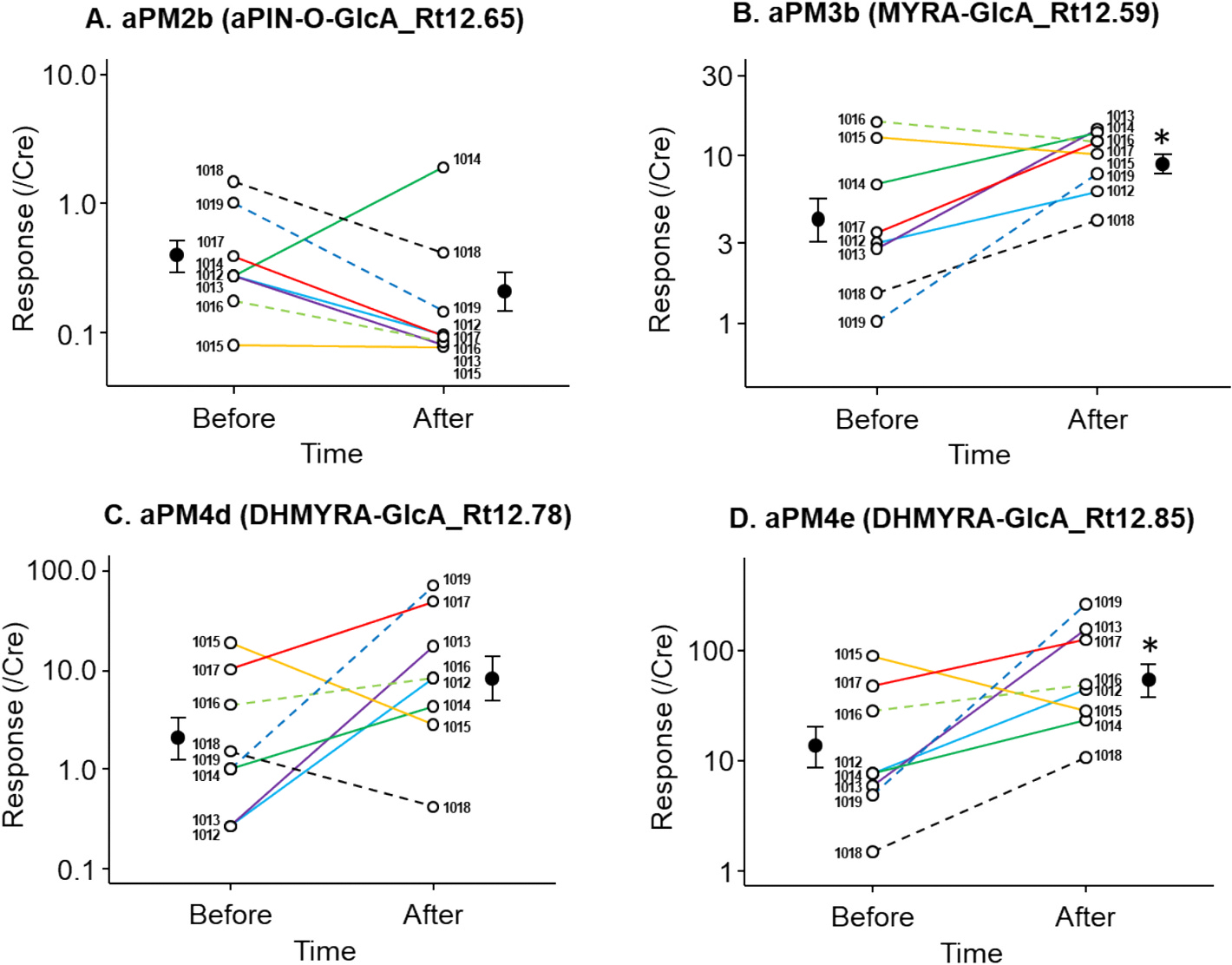
Levels of 4 abundant aPM in human urine collected before and after exposure to greenness. **A.** aPM2b (aPIN-O-GlcA_Rt12.65); **B**. aPM3b (MYRA-GlcA_Rt12.59); **C**. aPM4d (DHMYRA-GlcA_Rt12.78); **D**. aPM4e (DHMYRA-GlcA_Rt12.85). Human urine samples were collected before and after 4 h of forest bathing, and analyzed using UPLC-Q TOF-MS. For each aPM, peak responses were normalized to urinary creatinine levels, and plotted. The data points from each individual participant were connected with a line to show the trend over time. Filled circles and error bars: mean and SE; empty circles with lines: individual values (subject IDs labeled aside); solid lines: male subject; dashed lines: female subject. Student’s *t*-test was performed after log-transformation. (*: p < 0.05).

Out of the 22 aPM discovered following α-pinene inhalation, 13 were detected in urine samples collected after exposure to greenness (Table S7). The remaining 9 aPM, which were not detected, represent minor metabolites contributing less than 4 % to the overall aPM after α-pinene inhalation (Table S5). Despite a 24-h restriction from other α-pinene sources, baseline aPM levels were detectable in urine (Fig. 4). Notably, aPM3b, aPM4e, and aPM5g showed statistically significant changes after greenness exposure, and both aPM3b and aPM4e were also among the most abundant and analytically robust urinary aPM detected after α-pinene inhalation (Table S7). Fig. 4 shows that the levels of these two aPM increased significantly 4 h after exposure, suggesting their potential as biomarkers for greenness exposure. Representative chromatograms of these two metabolites before and after exposure are presented in Fig. S6. However, it is worth noting that the LC-MS peak of dihydromyrtenic acid-4-O-GlcA (aPM4e, DHMYRA-GlcA_Rt12.85), overlapped with a limonene metabolite dihydroperillic acid glucuronide (DHPA-GlcA_Rt12.85) we reported previously (Xie et al., 2024). Although derived from different parent compounds, these metabolites exhibit identical *m*/*z* (343.1398) and retention time (12.85 min), making it challenging to determine the precise contribution of α-pinene to this LC-MS peak.

The impact of high baseline urinary α-pinene metabolite levels was evaluated by testing whether exclusion of participants with the highest baseline values would reduce variability and affect statistical significance. As shown in Table S8, removing one or two individuals with the highest background values led to lower p-values for most metabolites. However, for three of the four major α-pinene metabolites examined (aPM2b, aPM3b, and aPM4e), these changes did not alter the outcome of the statistical test, and results remained either significant or non-significant as in the full dataset. The exception was aPM4d, where removal of one or two high-baseline participants lowered the p-value from 0.075 (not statistically significant) to 0.029 and 0.028 (statistically significant), respectively.

## 4. Discussion

To the best of our knowledge, this study represents the first investigation of human phase II metabolites following α-pinene inhalation and provides the first evidence of increased urinary aPM after real-world greenness exposure. Using ILGA and untargeted LC-MS workflows, we discovered 22 aPM in human urine, with nine novel structures annotated and two of them confirmed by synthetic standards. The discovery of these novel structures demonstrates gaps in knowledge of the phase II metabolism of monoterpenes. This study also presents the first known synthesis of glucuronide reference metabolites of α-pinene, specifically myrtenol glucuronide (MYR-GlcA, aPM2c) and verbenol glucuronide (VER-GlcA, aPM2b).

Following relative quantification of urinary aPM, we characterized their kinetic parameters to assess metabolic behavior. Analysis revealed aPM2b, aPM3b, aPM4e, aPM4d as the most abundant metabolites, suggesting their significant role in α-pinene metabolism (Tables 1 and 2). Notably, exposure to greenness was associated with significant elevations in aPM3b and aPM4e, suggesting these metabolites may serve as biomarkers for greenness exposure. The time course of elimination of the pinene metabolites described here will be important in designing future exposure studies, which should consider rates of elimination of metabolites, i.e., samples should be collected over time when the excretion of these metabolites is maximal (<1.5 h). In our study, the estimated Ke values for VER-GlcA (aPM2b), MYR-GlcA (aPM2c), and MYRA-GlcA (aPM3) ranged from 0.5 to 1.2 h^−1^, which are higher than the Ke values for related α-pinene metabolites (0.4–0.5 h^−1^) reported following oral exposure (Schmidt and Göen, 2017a). The different administration routes are unlikely to explain the discrepancy, as both lead to systemic absorption and renal clearance. A more likely explanation is the type of metabolites analyzed. Our study focused on glucuronidated forms, which are more hydrophilic than their unconjugated counterparts and are typically eliminated more rapidly via the kidneys. This difference in metabolite polarity and clearance pathway likely contributes to the higher elimination rates observed in our data.

Our results enable a direct comparison between the metabolism of α-pinene and limonene, two major monoterpenes widely emitted by plants (Bako et al., 2024; Geron et al., 2000; Yáñez-Serrano et al., 2018). The metabolic pathways of α-pinene (Fig. 1) and limonene (Fig. S1) exhibit notable similarities. Both monoterpenes, share the same molecular formula (C_10_H_16_) and monoisotopic molecule mass (136.1252 Da), and are oxidized to alcohols and acids during phase I metabolism and conjugated with glucuronic acid in phase II metabolism (Xie et al., 2024). This structural and metabolic similarity results in several metabolites with identical molecular weights and negative ion *m*/*z* values, potentially complicating analytical interpretation. Although chromatographic separation often resolves such isomers via retention time differences, overlaps may remain, particularly affecting the attribution of aPM4e. Specifically, peak 343.1398_12.85 corresponds to both a limonene metabolite (DHPA-GlcA_Rt12.85) and an aPM (DHMYRA-GlcA_Rt12.85, aPM4e). However, the shared connection of both metabolites to greenness exposure still allows their use as biomarkers. Observations of increased levels of this peak after exposure to forested areas (Fig. 4) validate its role as an indicator of greenness exposure.

The largest category of metabolites in the metabolic pathways of α-pinene and limonene are acid glucuronides, accounting for 16 of 22 aPM (Table 1) and 9 of 18 limonene metabolites (Xie et al., 2024). Acyl glucuronides, in particular, exhibit high reactivity, undergoing structural rearrangements that contribute to the formation of multiple isomers (Regan et al., 2010). This reactivity likely contributes to the extensive isomeric diversity observed in this study, as seen in DHMYRA (aPM4, 5 isomers) and MYRA-4-O-GlcA (aPM5, 7 isomers). Beyond posing an analytical challenge, acyl glucuronides can also have biological effects (Bailey and Dickinson, 2003; Bradshaw et al., 2020), because of their ability to form covalent adducts with proteins, which may affect biological activity and introduce toxicity.

The metabolism of α-pinene and limonene differs notably in the prevalence of alcohol glucuronides; with α-pinene yielding 5 (Table 1) and limonene 6 such metabolites (Xie et al., 2024). Limonene undergoes oxidation at multiple sites (Fig. S1), primarily at the exocyclic double bond, forming limonene-8,9-diol and its glucuronides, which constitute ~70 % of its urinary metabolites (Schmidt and Göen, 2017b; Xie et al., 2024). α-Pinene, lacking an exocyclic double bond, is oxidized only at the cyclohexenyl ring and methyl side-chain (Fig. 1), producing no diols (Schmidt and Göen, 2017a). As a result, only one of four most abundant aPM (aPM2b, VER-GlcA) is alcohol glucuronide, representing only ~ 17 % of total aPM (Table 2). This difference illustrates how structural variations between monoterpenes influence their metabolic fates.

Kinetic analysis of α-pinene (Table 2) and limonene metabolites (Xie et al., 2024) provided key insights into their potential as exposure biomarkers. Alcohol glucuronides generally exhibited faster generation (Ka) and elimination (Ke) rates than acid glucuronides. These kinetic patterns suggest that the ratio of alcohol to acid glucuronides could serve as an indicator of exposure timing. In our previous study (Xie et al., 2024) demonstrated this utility with limonene metabolites, using the ratio between LMN-8,9-O-GlcA (an alcohol glucuronide) and DHPA-GlcA (an acid glucuronide) to track recent exposure to greenness. However, no comparable trend was observed for aPM, likely due to the slower generation of α-pinene alcohol glucuronides. For limonene metabolites, Ka values for alcohol glucuronides ranged from 5 to 10 h^−1^, whereas α-pinene alcohol glucuronides were generated more slowly, with Ka values between 1.5 and 3.5 h^−1^. This difference aligns with limonene’s metabolic preference for producing alcohol glucuronides as dominant metabolites.

The elimination rates (Ke) for urinary metabolites are comparable for both monoterpenes, ranging from 0.46–1.4 h^−1^ for aPM and 0.25–0.99 h^−1^ for limonene metabolites, corresponding to half-lives (t_1/2_) of 0.5–1.5 h and 0.7–2.7 h, respectively (Table 2) (Xie et al., 2024). In contrast, blood half-lives (t_1/2α_) of α-pinene (~5 min) (Falk et al., 1990) and limonene (~3 min) (Falk-Filipsson et al., 1993) are extremely short, making them impractical for tracking exposure due to their rapid decline post-exposure. This likely accounts for prior inconsistent findings regarding blood monoterpenes as greenness markers (Bach et al., 2021; Sumitomo et al., 2015). Urinary metabolites, however, exhibit a more appropriate temporal profile, with significantly elevated levels still observed 4 h after exposure (Fig. 3). Therefore, urinary metabolites of α-pinene and limonene offer greater potential as biomarkers for greenness exposure than their parent compounds due to prolonged retention in the body. These results support the prioritization the measurements of urinary metabolite levels over blood concentrations of parent compounds in biomarker development for greenness-related studies in future.

α-Pinene in forest air consists of a mixture of (−) and (+) enantiomers in various ratios (Stephanou 2007). To simulate forest air, we used a racemic mixture for our human α-pinene inhalation experiment. However, it is beneficial to understand the contribution of each enantiomer to the formation of aPM. To achieve this, we conducted a murine exposure experiment using two different isomers. Mice, living in controlled environments with minimal background exposure, are ideal for such experiments (Jin et al., 2023). The results of this experiment can also confirm the origin of the aPM and provide information on species differences in α-pinene metabolism. Mouse urine data indicated that the two potential biomarkers for greenness (aPM3b and aPM4e; see Fig. 4) can be derived from both (−) and (+) α-pinene (Table S6). This is expected because the levels of aPM3b and aPM4e correlate with the total abundance of α-pinene of both enantiomers, enhancing sensitivity compared to more enantiomer-specific metabolites. However, more enantiomer-specific aPM probably can aid in identifying plant species associated with exposure, particularly coniferous trees (Allenspach and Steuer, 2021).

Although this study, along with previous research on limonene metabolism (Xie et al., 2024), lays a foundation for future investigations of additional bVOC metabolite biomarkers, it has certain limitations. First, of the 22 metabolites discovered in this study, only 9 were structurally assigned. Future investigations are necessary to complete this structural elucidation of the remaining metabolites, which may provide further insights into the metabolic pathways of α-pinene. Second, due to a lack of standards, the study used relative quantification of phase II metabolites of α-pinene. This approach revealed trends but not absolute levels of these metabolites, which are necessary to establish baseline levels and cut-off values for exposure to greenness. Third, the precision of pharmacokinetic estimates was constrained by the limited sample size, number of time points, and absence of urine volume measurements at each collection. The use of a pseudo–one-compartment model with first-order kinetics, while sufficient for describing general trends, remains a simplification of the true metabolic process. Fourth, we did not measure α-pinene concentrations in the ambient air during the greenness exposure study, which limited our ability to directly correlate environmental exposure with urinary biomarker levels. To provide context for interpreting our findings, we referenced published data on α-pinene concentrations and chirality in similar environments (Helmig and Arey 1992; Peters et al., 1994; Stephanou 2007). An additional limitation of this greenness exposure study is the relatively small sample size (n = 8), which restricts the statistical power and generalizability of our findings. As this was an exploratory analysis designed to identify and characterize candidate biomarkers, our conclusions are preliminary and should be validated in larger, more diverse cohorts in future studies. Finally, the study did not investigate sources of α-pinene other than greenness exposure. Similar to previous research on limonene exposure (Xie et al., 2024), significant variation in aPM levels is observed prior to greenness exposure (Fig. 4). This suggests that aPM alone may not be sufficiently specific as a biomarker for greenness exposure. In human biomonitoring studies, other sources of α-pinene such as food, personal care products, and household products, cannot be excluded. Therefore, combining measurement of urinary metabolites with data from questionnaires, interviews, Normalized Difference Vegetation Index (NDVI), or GPS data reflecting physical activity around greenspaces would likely provide a more comprehensive assessment of individual exposures.

## 5. Conclusions

In this study, 22 human urinary metabolites with 9 novel structures were discovered following α-pinene inhalation, and their kinetics were analyzed. We also examined the relationships between real world greenness exposure and urinary metabolite levels of aPM. Based on these findings, we propose that myrtenic acid glucuronide (MYRA-GlcA) and dihydromyrtenic acid glucuronide (DHMYRA-GlcA) may serve as individual-level markers of greenness exposure when used alongside other complementary study tools. The discovery of new aPM enhances our understanding of monoterpenoid metabolism in humans and facilitates the study of the beneficial effects of greenness exposure at an individual level.

## Supplementary Material

1

Appendix A. Supplementary data

Supplementary data to this article can be found online at https://doi.org/10.1016/j.envint.2025.109710.

## Figures and Tables

**Table 1:** Peak assignments of α-pinene metabolites (aPM) in human urine after inhalation of the parent compound.

Metabolite ID	LC-MS peak (*m/z*_Rt)	Peak selection method	Number of peaks with same *m/z*	Number of library items with matching *m/z*	Assignment rules applied	Assigned peak identity*

aPM1	179.0714_9.91	Untargeted	1	0	(5)	MYRAO
aPM2a	327.1449_12.50	ILGA	3	3	(3b) + (2)	aPIN-O-GlcA_Rt12.50
aPM2b	327.1449_12.65	Both	3	3	(3b) + (2)	VER-GlcA**
aPM2c	327.1449_12.83	Both	3	3	(3b) + (2)	MYR-GlcA**
aPM3a	341.1242_7.37	Both	2	2	(3a) + (2)	MYRA-GlcA_Rt7.37
aPM3b	341.1242_12.59	Both	2	2	(3a) + (2)	MYRA-GlcA_Rt12.59
aPM4a	343.1398_7.98	Both	5	2	(3a) + (2)	DHMYRA-GlcA_Rt7.98
aPM4b	343.1398_8.85	Both	5	2	(3a) + (2)	DHMYRA-GlcA_Rt8.85
aPM4c	343.1398_10.99	ILGA	5	2	(3a) + (2)	DHMYRA-GlcA_Rt10.99
aPM4d	343.1398_12.78	ILGA	5	2	(3a) + (2)	DHMYRA-GlcA_Rt12.78
aPM4e	343.1398_12.85	Both	5	2	(3a) + (2)	DHMYRA-GlcA_Rt12.85
aPM5a	357.1191_5.05	ILGA	7	1	(2)	MYRA-4-O-GlcA_Rt5.05
aPM5b	357.1191_6.47	ILGA	7	1	(2)	MYRA-4-O-GlcA_Rt6.47
aPM5c	357.1191_6.53	ILGA	7	1	(2)	MYRA-4-O-GlcA_Rt6.53
aPM5d	357.1191_6.83	ILGA	7	1	(2)	MYRA-4-O-GlcA_Rt6.83
aPM5e	357.1191_7.63	ILGA	7	1	(2)	MYRA-4-O-GlcA_Rt7.63
aPM5f	357.1191_7.79	ILGA	7	1	(2)	MYRA-4-O-GlcA_Rt7.79
aPM5g	357.1191_8.14	Both	7	1	(2)	MYRA-4-O-GlcA_Rt8.14
aPM6	359.1348_7.41	Untargeted	1	0	(5)	DHMYRA-4-O-GlcA
aPM7a	361.1504_4.85	Untargeted	2	0	(5) + (2)	PNRL-GlcA_Rt4.85
aPM7b	361.1504_5.27	Untargeted	2	0	(5) + (2)	PNRL-GlcA_Rt5.27
aPM8	373.1140_3.10	Untargeted	1	0	(5)	MYRA-4-O-7-O-GlcA

* Full names of abbreviations: MYRAO: Myrtenic acid-4-one; aPIN-O-GlcA: α-Pinene-O-GlcA; VER-GlcA: Myrtenol-GlcA; VER-GlcA: Verbenol-GlcA; MYRA-GlcA: Myrtenic acid-GlcA; DHMYRA-GlcA: Dihydromyrtenic acid-GlcA; MYRA-4-O-GlcA: Myrtenic acid-4-O-GlcA; DHMYRA-4-O-GlcA: Dihydromyrtenic acid-4-O-GlcA; PNRL-GlcA: Pinanetriol-GlcA; MYRA-4-O-7-O-GlcA: Myrtenic acid-4-O-7-O-GlcA.

** The initial identities assigned to the peaks were aPIN-O-GlcA_Rt12.65 and aPIN-O-GlcA_Rt12.83. Subsequent confirmation identified the corresponding metabolites as VER-GlcA and MYR-GlcA through LC-MS/MS analysis with synthesized compounds.

**Table 2:** Estimation of kinetic parameters for urinary aPM with a pseudo one-compartment model.

Metabolite ID	F (%) *	Ka (h^−1^)	Observed T_max_ (h)	Ke (h^−1^)	Elimination t_1/2_(h)

aPM1	1.15 ± 0.39	1.25 ± 0.43	1	0.58 ± 0.18	1.20 ± 0.38
aPM2a	0.51 ± 0.12	2.69 ± 0.68	0.5	1.23 ± 0.17	0.56 ± 0.08
aPM2b	17.23 ± 3.91	3.46 ± 0.98	0.5	1.17 ± 0.15	0.59 ± 0.08
aPM2c	6.99 ± 2.12	3.02 ± 0.88	0.5	1.39 ± 0.26	0.50 ± 0.09
aPM3a	2.92 ± 0.94	1.80 ± 0.67	1	0.75 ± 0.22	0.93 ± 0.27
aPM3b	22.95 ± 8.38	1.98 ± 0.60	1	1.13 ± 0.24	0.61 ± 0.13
aPM4a	3.19 ± 1.11	2.12 ± 0.87	1	0.98 ± 0.30	0.71 ± 0.22
aPM4b	1.47 ± 0.63	1.34 ± 0.47	1	0.87 ± 0.28	0.79 ± 0.26
aPM4c	0.26 ± 0.08	1.65 ± 0.67	1	0.46 ± 0.16	1.51 ± 0.54
aPM4d	13.03 ± 5.57	0.73 ± 0.29	1	0.68 ± 0.27	1.01 ± 0.40
aPM4e	18.14 ± 6.52	1.26 ± 0.50	1	0.70 ± 0.24	0.99 ± 0.33
aPM5a	0.25 ± 0.09	1.64 ± 0.77	1	0.70 ± 0.27	1.00 ± 0.38
aPM5b	1.46 ± 0.57	1.74 ± 0.84	1	0.80 ± 0.31	0.87 ± 0.34
aPM5c	1.28 ± 0.53	1.73 ± 0.89	1	0.79 ± 0.32	0.88 ± 0.36
aPM5d	0.27 ± 0.09	1.33 ± 0.51	1	0.53 ± 0.19	1.31 ± 0.46
aPM5e	0.38 ± 0.16	0.91 ± 0.36	1	0.91 ± 0.34	0.76 ± 0.28
aPM5f	0.29 ± 0.12	1.53 ± 0.71	1	0.73 ± 0.29	0.95 ± 0.37
aPM5g	5.10 ± 2.25	1.62 ± 0.81	1	1.01 ± 0.43	0.69 ± 0.29
aPM6	0.67 ± 0.27	1.87 ± 1.09	1	0.53 ± 0.24	1.31 ± 0.59
aPM7a	1.56 ± 0.66	1.46 ± 0.48	1	0.93 ± 0.27	0.74 ± 0.22
aPM7b	0.28 ± 0.09	2.69 ± 0.73	0.5	1.15 ± 0.22	0.61 ± 0.12
aPM8	0.65 ± 0.31	0.73 ± 0.33	1	0.71 ± 0.31	0.97 ± 0.42

* F values were normalized to the sum of 22 detected metabolites.

## Data Availability

Data will be made available on request.

## Abbreviations:

bVOC: biogenic volatile organic compounds
ILGA: integrated library-guided analysis
UPLC-QTOF-MS: ultra-performance liquid chromatography-quadrupole time-of-flight mass spectrometry
DIA: data-independent acquisition
aPM: α-pinene metabolite(s)
NDVI: Normalized Difference Vegetation Index
Rt: retention time
NLME: nonlinear mixed effect model.
