# Supplementary material for: Human exposure to green spaces and monoterpenes: discovery and use of α-pinene metabolites as biomarkers
Source: Environ Int. Author manuscript; Available in PMC 2025 Sep 29. (PMC12477777; doi:10.1016/j.envint.2025.109710)
Supplement: 1 [file NIHMS2105995-supplement-1.docx]

**Human Exposure to Green Spaces and Biogenic Volatile Organic Compounds: Discovery and Use of α-Pinene Metabolites as Biomarkers**

Zhengzhi Xie ^a,b,c,e^, Saurin R. Sutaria ^a,b,c,d,e^, Jin Y. Chen ^a,b,c,e^, Hong Gao ^a,b,c,e^, Daniel J. Conklin^a,b,c,e^, Rachel J. Keith^a,b,c,e^, Sanjay Srivastava^a,b,c,e^, Pawel Lorkiewicz*^a,b,c,e,f^, and Aruni Bhatnagar^a,b,c,e^

^a^Christina Lee Brown Envirome Institute, University of Louisville;

^b^Superfund Research Center, University of Louisville;

^c^American Heart Association-Tobacco Regulation and Addiction Center, University of Louisville;

^d^Department of Chemistry, Bellarmine University;

^e^Division of Environmental Medicine, Department of Medicine, University of Louisville, Louisville, KY 40202, USA; and

^f^Department of Chemistry, University of Louisville;

**Supporting Information (SI)**

Number of pages: 33

Number of tables: 8

Number of figures: 8

***aPM discovery and identification***

Two complementary methods, ILGA and an untargeted approach, were employed to identify candidate aPM peaks from raw LC-MS data. Our group developed the ILGA workflow for profiling and pseudo-targeted analysis of phase II metabolites in urine [1], and we used it to identify and quantify limonene metabolites [2]. To complement the ILGA workflow, which requires pre-knowledge of α-pinene metabolites (aPM), we also used an unbiased LC-MS-based untargeted analysis. This untargeted approach follows a well-established workflow for biomarker discovery [3-7]. For this study, we chose Progenesis QI for spectral deconvolution and peak alignment for untargeted data processing [6, 7]. This combined approach discovered 22 aPM peaks. Of these, 9 were found exclusively through the ILGA workflow, 8 by both methods, and 5 solely through untargeted analysis (**Table 1**). These 5 peaks were associated with 4 distinct *m/z* values which represented half of the 8 unique *m/z* values discovered in this study, demonstrating the capability of the untargeted approach and the value of combining both metabolite discovery workflows.

Using ILGA, seventeen candidate peaks, with four distinct m/z values, were discovered and their identities were assigned according to a pre-established library, as previously reported [2] (**Table 1** and **S3**). In addition, isomers were identified for each peak, and retention times (Rt) were used to supplement their assignments to distinguish the isomers according to rule (2). Notably, although all these peaks were assigned as glucuronides previously unreported, their aglycones have been reported in human urine [8], and thus these aPMs are only “half-unknown”.

- aPM2 exhibited three peaks at m/z 327.1449, which matched MYR-GlcA and VER-GlcA in the library. These peaks shared similar MS/MS spectra (**Figure 2A, 2C,** and **S7A**), featuring a glucuronide moiety (m/z 175.02) with fragment ions at m/z 113.02, 99.00, 95.01, 85.02, 75.00 [9, 10]. The fragment at m/z 309.13 indicated a water loss ([M-H-H_2_O]), while an aglycone fragment at m/z 151.11, with an extra hydroxy group compared to α-pinene, again, suggested MYR or VER. Both compounds are known urinary metabolites capable of glucuronide conjugation [8]. Therefore, peaks of aPM2 were assigned as aPIN-O-GlcA under rule (3b).
- aPM3: The two peaks of aPM3 corresponded to two compounds in the library: MYRA-GlcA and MYL-4-O-GlcA. Given that the aglycone MYRA has been previously detected in human urine [8], aPM3 was identified as MYRA-GlcA following rule (3a).
- aPM4: The five peaks of aPM4 matched two items in the library (DHMYRA-GlcA and MYR-4-O-GlcA). As the aglycone DHMYRA has been found in human urine [8], aPM4 was identified as DHMYRA-GlcA according to rule (3a).
- aPM5: The seven peaks of aPM5 (m/z: 357.1191) correspond to only one item in the library (**Table S4**), and based on rule (1), they have been identified as MYRA-4-O-GlcA.

The other five peaks, with four distinct m/z values, were discovered exclusively using the untargeted method (**Table 1**). Assigning identities to the peaks discovered via untargeted analysis poses a greater challenge. Unlike ILGA-discovered features, these peaks lack direct links to established metabolite identities in the scouting library. Nonetheless, considering the shared origin of all metabolites from a common parent compound, it is logical to propose that their structures are related, and their levels are correlated. The process of assigning putative identities to unknown peaks has been observed to be facilitated through the correlation of their levels with identified metabolites [11, 12]. Consequently, we adjusted the previous rules to accommodate findings from untargeted analysis (see **Figure S3**). Specifically, a new rule (5) was introduced, proposing a new structure for the unknown peak based on its mass shifts in relation to identified metabolites displaying strong correlation in levels (**Figure S8**). In the following part of this section, new structures were assigned to these peaks according to rule (5), aided by correlation data presented in **Figure S8** and the mass shifts related to the aPMs assigned in the previous section.

- aPM1: The correlation heatmap (**Figure S8**) revealed that aPM1 was clustered with glucuronides of MYRA-4-OH (aPM5) and DHMYRA (aPM4), suggesting a shared metabolic pathway for these metabolites. The [M-H]^-^ ion for aPM1 (m/z 179.0714) is 2.0156 Da lighter than that of MYRA-4-OH (m/z 181.0870), implying the loss of two hydrogen atoms. Consequently, aPM1 was identified as MYRAO, an aldehyde probably formed from the alcohol MYRA-4-OH through the removal of two hydrogens (**Figure 1**).
- aPM6: This peak showed a strong correlation with glucuronides of MYRA-4-OH, MYRA, and DHMYRA (**Figure S8**), indicating a possible structural similarity with these metabolites. Its ion (m/z 359.1348) is 2.0157 Da heavier than MYRA-4-O-GlcA (m/z 357.1191), or 15.995 Da heavier than DHMYRA-GlcA (m/z 343.1398) suggesting an addition of two hydrogen atoms on MYRA-4-O-GlcA or one oxygen atom on DHMYRA-GlcA. Therefore, aPM6 was identified as DHMYRA-4-O-GlcA. Its aglycone, DHMYRA-4-OH, is probably formed from MYRA-4-OH by the addition of two hydrogen atoms, or from DHMYRA by addition of one oxygen atom (**Figure 1**).
- aPM7: The two peaks of aPM7, particularly the one at Rt 5.27, showed a strong correlation with the VER-GlcA and MYR-GlcA (**Figure S8**). This suggests a connection between aPM7 and aPIN-OH (MYR or VER). The m/z value of aPM7, 361.1504, is 34.0055 Da heavier than the aPIN-O-GlcA ion (m/z 327.1449). This mass increase corresponds to the addition of two oxygen and two hydrogen atoms (**Figure 1**). Therefore, aPM7 was identified as [aPIN +3O +2H]-GlcA. One possible structure fit this identity is PNRL-GlcA. The aglycone of this metabolite, pinanetriol (PNRL) has been reported in *Tithonia diversifolia* [13]. Consequently, aPM7 was tentatively identified as PNRL-GlcA. The retention times (Rt) of aPM7 (4.85 and 5.27) were included in the label to differentiate the two isomers in accordance with rule (2).
- aPM8: This peak showed a strong correlation with MYRA-4-O-GlcA (aPM5a) and DHMYRA-4-O-GlcA (aPM6) (**Figure S8**), suggesting structural similarities among these metabolites. The m/z of aPM7 was 373.1140, which is 15.9949 Da higher than that of MYRA-4-O-GlcA (m/z 357.1191), implying the addition of an oxygen atom (**Figure 1**). Therefore, aPM8 was identified as [MYRA-4-O+O]-GlcA. One possible structure fit this identity is MYRA-4-O-7-O-GlcA. Consequently, aPM8 was tentatively identified as MYRA-4-O-7-O-GlcA.

After the identification, structures of the four most abundant aPMs were validated by LC-MS/MS (**Figure 2** and **S7**). MS/MS spectra were collected around the Rt of these peaks, and then inspected for the feature of both glucuronide moiety and the aglycone of the metabolites. Structure of aPM2 (three metabolites, see **Table 1**), with m/z matching MYR-GlcA and VER-GlcA, were further confirmed with comparative LC-MS/MS analyses with synthesized compounds.

- Comparative LC-MS/MS analyses of aPM2b with synthesized VER-GlcA confirmed a match in retention time (Rt) and spectra (**Figures 2A** and **2B**). Similarly, aPM2c matched MYR-GlcA in Rt and spectra (**Figures 2C** and **2D**). Although the Rt of aPM2a did not align with either standard, its MS/MS spectrum (**Figure S7A**) indicated structural similarity, suggesting a glucuronide with a related aglycone, possibly an enantiomer of VER or MYR. aPM2a was identified as aPIN-O-GlcA_Rt12.50 under rule (3b) and labeled with its retention time for precise identification (**Table 1**)
- The structure of aPM3b was confirmed based on its MS/MS spectrum. The MS/MS spectrum (**Figure S7B**) showed the presence of a glucuronide moiety at m/z 193.03 and 175.02 [14, 15] and fragment ions at 113.02, 99.00, 95.01, 85.02, and 75.00 [9, 10]. The MS/MS peak at 165.09, representing the aglycone form of the molecule, indicated an additional oxygen with a double bond compared to the aglycone peak of aPM2b at 151.1101. This aglycone could be MYRA, which is present in human urine [8] and can conjugate with glucuronide. Thus, aPM3b was confirmed as MYRA-GlcA.
- The structure of aPM4d and aPM4e was confirmed based on their MS/MS spectrum (**Figure S7C** and **S7D**). The MS/MS spectra of both compounds were very similar, indicating similar structures for these isomers. Both spectra featured a glucuronide moiety at m/z 193.03 and 175.02 [14, 15] with fragment ions at 113.02, 99.00, 95.01, 85.02, and 75.00 / 75.01 [9, 10]. The base peak at 167.10, representing the aglycone form, indicated an additional two hydrogens compared to aPM3b's aglycone peak at 165.09 (**Figure S7B**). This aglycone could be DHMYRA, present in human urine [8] and can conjugate with glucuronide. Consequently, aPM4d and aPM4e were confirmed as DHMYRA-GlcA, with Rt values in their acronyms for differentiation with other isomers (**Table 1**).

All phase II aPM were identified as glucuronides. Sulfates, glycine conjugates, and taurine conjugates were not found despite their inclusion in the scouting library (**Table S3**) and in a pseudo-targeted search using the ILGA approach. Our earlier work reported that glucuronides are the predominant phase II metabolites in limonene metabolism (Xie et al., 2024). Together, these discoveries suggest that glucuronidation is the main phase II metabolic pathway after monoterpene inhalation.

**Table S1:** Demographic characteristics of study participants.

| **Variable** | **α-Pinene inhalation** | **Greenness exposure** |
| --- | --- | --- |
|  | **(n = 8)** | **(n = 8)** |
| Sex, Male | 4 (50%) | 5 (60%) |
| Age Range | 25-50 | 25-70 |
| Race |  |  |
| White | 6 (75%) | 7 (87.5%) |
| Black | 0 (0%) | 1 (12.5%) |
| Other | 2 (25%) | 0 (0%) |

**Table S2**. Retention time shifts and mass errors of α-pinene metabolites (aPM) found in QC samples.

| Metabolite | Theoretical m/z | Mass error (mDa) | | | | | | Average Rt (min) | Rt shift (min) | | | | | |
| --- | --- | --- | --- | --- | --- | --- | --- | --- | --- | --- | --- | --- | --- | --- |
|  |  | QC1 | QC2 | QC3 | QC4 | QC5 | QC6 |  | QC1 | QC2 | QC3 | QC4 | QC5 | QC6 |
| aPM1 | 179.0714 | -0.6 | -0.4 | -0.4 | -0.2 | -0.4 | 0.0 | 9.91 | 0.00 | 0.01 | 0.01 | 0.00 | 0.01 | 0.00 |
| aPM2a | 327.1449 | -0.2 | -0.3 | 0.6 | -0.2 | 0.0 | -0.4 | 12.5 | 0.01 | 0.01 | 0.01 | 0.01 | 0.01 | 0.00 |
| aPM2b | 327.1449 | -0.3 | -0.7 | -0.1 | -0.2 | -0.3 | -0.1 | 12.65 | 0.00 | 0.00 | 0.00 | 0.00 | 0.00 | 0.00 |
| aPM2c | 327.1449 | -0.7 | -0.5 | -0.5 | -0.5 | -0.2 | -0.2 | 12.83 | 0.01 | 0.01 | 0.01 | 0.01 | 0.01 | 0.00 |
| aPM3a | 341.1242 | -0.6 | -0.4 | -0.2 | -0.6 | -0.2 | -0.2 | 7.37 | 0.00 | 0.00 | 0.00 | 0.00 | 0.00 | 0.00 |
| aPM3b | 341.1242 | -0.4 | -0.5 | -0.2 | -0.2 | -0.4 | -0.3 | 12.59 | 0.01 | 0.01 | 0.01 | 0.01 | 0.01 | 0.00 |
| aPM4a | 343.1398 | -0.7 | -0.4 | -0.4 | -0.3 | -0.3 | -0.1 | 7.98 | 0.00 | 0.00 | 0.00 | 0.00 | 0.00 | 0.00 |
| aPM4b | 343.1398 | -0.7 | -0.7 | -0.5 | 0.3 | -0.6 | 0.3 | 8.85 | 0.01 | 0.01 | 0.01 | 0.00 | 0.01 | 0.00 |
| aPM4c | 343.1398 | -0.4 | -0.4 | -0.1 | 0.2 | 0.1 | 0.1 | 10.99 | 0.00 | 0.00 | 0.01 | 0.00 | 0.01 | 0.00 |
| aPM4d | 343.1398 | -0.2 | -0.3 | -0.1 | 0.2 | -0.1 | -0.1 | 12.78 | 0.00 | 0.00 | 0.01 | 0.00 | 0.01 | 0.00 |
| aPM4e | 343.1398 | -0.4 | -0.3 | -0.2 | -0.1 | -0.1 | 0.0 | 12.85 | 0.00 | 0.01 | 0.01 | 0.00 | 0.01 | 0.00 |
| aPM5a | 357.1191 | -0.6 | 0.1 | 0.1 | -1.2 | 0.6 | 0.0 | 5.05 | 0.00 | 0.00 | 0.00 | 0.00 | 0.00 | 0.00 |
| aPM5b | 357.1191 | 1.1 | 0.8 | 0.7 | 0.8 | 0.9 | 1.5 | 6.47 | 0.00 | 0.01 | 0.01 | 0.01 | 0.01 | 0.01 |
| aPM5c | 357.1191 | 0.2 | 0.8 | 0.0 | 0.8 | 0.0 | 1.4 | 6.53 | 0.00 | 0.01 | 0.01 | 0.01 | 0.01 | 0.01 |
| aPM5d | 357.1191 | -0.5 | -0.2 | 0.4 | 0.0 | 0.2 | 0.5 | 6.83 | 0.00 | 0.01 | 0.01 | 0.01 | 0.01 | 0.01 |
| aPM5e | 357.1191 | -0.4 | 0.5 | -0.2 | 1.4 | -0.4 | 1.1 | 7.63 | 0.00 | 0.00 | 0.00 | 0.00 | 0.00 | 0.00 |
| aPM5f | 357.1191 | -0.2 | -0.9 | -0.5 | 0.0 | 0.6 | -0.6 | 7.79 | -0.01 | 0.00 | 0.00 | 0.00 | 0.01 | 0.00 |
| aPM5g | 357.1191 | -0.7 | -0.2 | -0.4 | -0.4 | -0.6 | 0.1 | 8.14 | -0.01 | 0.00 | 0.00 | 0.00 | 0.00 | 0.00 |
| aPM6 | 359.1348 | 0.0 | -0.2 | -0.4 | 0.0 | -0.1 | 0.2 | 7.41 | -0.02 | -0.01 | -0.01 | -0.01 | -0.01 | -0.01 |
| aPM7a | 361.1504 | -0.2 | -0.3 | -0.5 | -0.8 | -1.3 | -0.4 | 4.85 | 0.00 | 0.00 | 0.00 | 0.00 | 0.00 | 0.00 |
| aPM7b | 361.1504 | -0.3 | -0.5 | -0.1 | -1.6 | -2.5 | 0.9 | 5.27 | -0.02 | -0.02 | -0.02 | -0.02 | -0.02 | -0.02 |
| aPM8 | 373.114 | -1.2 | 0.1 | -0.4 | -0.4 | 0.7 | 0.2 | 3.1 | 0.00 | 0.00 | 0.00 | 0.00 | 0.00 | 0.00 |

**Table S3**: Full list of known and proposed aPM included in the scouting library

| **Full name^*^** | **Acronym** | **SMILES** | **CAS Number ^**^** | **Formula** | **Monoisotopic mass** | **Theoretical m/z [M-H]^-^ ^***^** | **Rt (min)** | **Ref** |
| --- | --- | --- | --- | --- | --- | --- | --- | --- |
| α-Pinene | aPIN | C1=C(C)C2CC(C1)C2(C)C | 80-56-8 | C10H16 | 136.1252 | 135.1179 |  |  |
| α-Pinene-2,3-epox | aP23ox | CC12C(C3)C(C)(C)C3CC1O2 | 1686-14-2 | C10H16O | 152.1201 | 151.1128 |  | [16] |
| Dihydromyrtenic acid | DHMYRA | CC1(C)C2C(CCC1C2)C(O)=O | 76198-24-8 | C10H16O2 | 168.115 | 167.1078 |  | [8] |
| Dihydromyrtenic acid-GlcA | DHMYRA-GlcA | CC1(C)C2C(CCC1C2)C(OC3C(O)C(O)C(O)C(C(O)=O)O3)=O |  | C16H24O8 | 344.1471 | 343.1396 | 7.98, 8.85, 10.99, 12.78, 12.85 |  |
| Dihydromyrtenic acid-Gly | DHMYRA-Gly | CC1(C)C2C(CCC1C2)C(NCC(O)=O)=O |  | C12H19NO3 | 225.1365 | 224.1292 |  |  |
| Dihydromyrtenic acid-Sulfate | DHMYRA-SA | CC1(C)C2C(CCC1C2)C(OS(=O)(O)=O)=O |  | C10H16O5S | 248.0718 | 247.0646 |  |  |
| Dihydromyrtenic acid-Taur | DHMYRA-T | CC1(C)C2C(CCC1C2)C(NCCS(=O)(O)=O)=O |  | C12H21NO4S | 275.1191 | 274.1119 |  |  |
|  |  |  |  |  |  |  |  |  |
|  |  |  |  |  |  |  |  |  |
| Myrtenal-4-OH | MYL-4-OH | CC1(C)C2C(C(O)=O)=CC(O)C1C2 | 175892-11-2 | C10H14O2 | 166.0994 | 165.0921 |  | [17] |
| Myrtenal-4-O-GlcA | MYL-4-O-GlcA | CC1(C)C2C(C=O)=CC(OC3C(O)C(O)C(O)C(C(O)=O)O3)C1C2 |  | C16H22O8 | 342.1315 | 341.1242 |  |  |
| Myrtenal-4-O-Sulfate | MYL-4-SA | CC1(C)C2C(C=O)=CC(OS(=O)(O)=O)C1C2 |  | C10H14O5S | 246.0562 | 245.0489 |  |  |
| Myrtenol | MYR | CC1(C)C2C(CO)=CCC1C2 | 515-00-4 | C10H16O | 152.1201 | 151.1128 |  | [8] [18] [19] |
| Myrtenol-GlcA | MYR-GlcA | CC1(C)C2C(COC3OC(C(O)=O)C(O)C(O)C3O)=CCC1C2 |  | C16H24O7 | 328.1522 | 327.1449 | 12.50, 12.65, 12.83 |  |
| Myrtenol-Sulfate | MYR-SA | CC1(C)C2C(COS(=O)(O)=O)=CCC1C2 |  | C10H16O4S | 232.0769 | 231.0697 |  |  |
| Myrtenol-4-OH | MYR-4-OH | CC1(C)C2C(CO)=CC(O)C1C2 | 1823934-93-5 | C10H16O2 | 168.115 | 167.1078 |  | [17] |
| Myrtenol-4-O-GlcA | MYR-4-O-GlcA | CC1(C)C2C(COC3OC(C(O)=O)C(O)C(O)C3O)=CC(O)C1C2 |  | C16H24O8 | 344.1471 | 343.1398 |  |  |
| Myrtenol-4-O-Sulfate | MYR-4-O-SA | CC1(C)C2C(COS(O)(=O)=O)=CC(O)C1C2 |  | C10H16O5S | 248.0718 | 247.0646 |  |  |
| Myrtenic acid | MYRA | CC1(C)C2C(C(O)=O)=CCC1C2 | 19250-17-0 | C10H14O2 | 166.0994 | 165.0921 |  | [8] [19] [20] |
| Myrtenic acid-GlcA | MYRA-GlcA | CC1(C)C2C(C(OC3C(O)C(O)C(O)C(C(O)=O)O3)=O)=CCC1C2 |  | C16H22O8 | 342.1315 | 341.1242 | 7.37, 12.59 |  |
| Myrtenic acid-Gly | MYRA-Gly | CC1(C)C2C(C(NCC(O)=O)=O)=CCC1C2 |  | C12H17NO3 | 223.1208 | 222.1136 |  |  |
| Myrtenic acid-Sulfate | MYRA-SA | CC1(C)C2C(C(OS(=O)(O)=O)=O)=CCC1C2 |  | C10H14O5S | 246.0562 | 245.0489 |  |  |
| Myrtenic acid-Taur | MYRA-T | CC1(C)C2C(C(NCCS(=O)(O)=O)=O)=CCC1C2 |  | C12H19NO4S | 273.1035 | 272.0962 |  |  |
| Myrtenic acid-4-OH | MYRA-4-OH | CC1(C)C2C(C(O)=O)=CC(O)C1C2 | 82276-79-7 | C10H14O3 | 182.0943 | 181.087 |  | [8] |
| Myrtenic acid-4-O-GlcA | MYRA-4-O-GlcA | CC1(C)C2C(C(O)=O)=CC(OC3C(O)C(O)C(O)C(C(O)=O)O3)C1C2 |  | C16H22O9 | 358.1264 | 357.1191 | 5.05, 6.47, 6.53, 6.83, 7.63, 7.79, 8.14 |  |
| Myrtenic acid-4-O-Sulfate | MYRA-4-O-SA | CC1(C)C2C(C(O)=O)=CC(OS(O)(=O)=O)C1C2 |  | C10H14O6S | 262.0511 | 261.0438 |  |  |
| Myrtenic acid-4-N-Taur | MYRA-4-N-T | CC1(C)C2C(C(NCCS(O)(=O)=O)=O)=CC(O)C1C2 |  | C12H19NO5S | 289.0984 | 288.0911 |  |  |
| Myrtenic acid-4-OH-Gly | MYRA-4-OH-Gly | CC1(C)C2C(C(NCC(O)=O)=O)=CC(O)C1C2 |  | C12H17NO4 | 239.1158 | 238.1085 |  |  |
| Verbenol | VER | CC1=CC(O)C2C(C)(C)C1C2 | 473-67-6 | C10H16O | 152.1201 | 151.1128 |  | [8] [17] [19] [20] [18] |
| Verbenol-GlcA | VER-GlcA | CC1=CC(OC2C(O)C(O)C(O)C(C(O)=O)O2)C3C(C)(C)C1C3 |  | C16H24O7 | 328.1522 | 327.1449 | 12.50, 12.65, 12.83 |  |
| Verbenol-Sulfate | VER-SA | CC1=CC(OS(=O)(O)=O)C2C(C)(C)C1C2 |  | C10H16O4S | 232.0769 | 231.0697 |  |  |

* This table presents 29 aPMs from the scouting library (rows with no background color) and 7 aPMs not included in the scouting library (rows with a gray background). The 7 aPMs comprise 4 phase II aPMs discovered through untarget analysis and 3 phase I aPMs linked to these 4 metabolites.

**Among the 29 metabolites in the scouting library, 9 with CAS Registry Numbers are reported aPMs, while the remaining 20 metabolites are proposed aPMs. Of the 7 aPMs not included in the scouting library, 2 with CAS Registry Numbers have only been identified as secondary metabolites in plant extracts and have never been found in animals or humans as aPM.

***Theoretical m/z [M-H] ^-^ shown in grey are not likely to ionize in ESI

**Table S4**: aPM found by untargeted analysis

| **Full name^*^** | **Acronym** | **SMILES** | **CAS Number ^**^** | **Formula** | **Monoisotopic mass** | **Theoretical m/z [M-H]^-^ ^***^** | **Rt (min)** | **Ref** |
| --- | --- | --- | --- | --- | --- | --- | --- | --- |
| Dihydromyrtenic acid-4-OH | DHMYRA-4-OH | CC1(C)C2C(C(O)=O)CC(O)C1C2 |  | C10H16O3 | 184.1099 | 183.1027 |  |  |
| Dihydromyrtenic acid-4-O-GlcA | DHMYRA-4-O-GlcA | CC1(C)C2C(C(O)=O)CC(OC3C(O)C(O)C(O)C(C(O)=O)O3)C1C2 |  | C16H24O9 | 360.142 | 359.1348 | 7.41 |  |
| Myrtenic acid-4-O-7-OH | MYRA-4-O-7-OH | CC1(C)C2(O)C(C(O)=O)=CC(O)C1C2 |  | C10H14O4 | 198.0892 | 197.0819 |  |  |
| Myrtenic acid-4-O-7-O-GlcA | MYRA-4-O-7-O-GlcA | CC1(C)C2(O)C(C(O)=O)=CC(OC3C(O)C(O)C(O)C(C(O)=O)O3)C1C2 |  | C16H22O10 | 374.1213 | 373.114 | 3.1 |  |
| Myrtenic acid-4-one | MYRAO | CC1(C)C2C(C(O)=O)=CC(C1C2)=O | 2652000-66-1 | C10H12O3 | 180.0786 | 179.0714 | 9.91 | [21] |
| Pinanetriol | PNRL | OC1(CO)C2C(C)(C)C(C2)CC1O | 1610877-05-8 | C10H18O3 | 186.1256 | 185.1183 |  | [13] |
| Pinanetriol-GlcA | PNRL-GlcA | OC1(COC2C(O)C(O)C(O)C(C(O)=O)O2)C3C(C)(C)C(C3)CC1O |  | C16H26O9 | 362.1577 | 361.1504 | 4.85, 5.27 |  |

**Table S5.** Response of aPM in human urine after α-pinene inhalation

| Metabolite ID | LC-MS peak  (m/z_Rt) | p-value* | Frequency of occurrence | Maximum response | Proportion (observed) after  α-pinene inhalation |
| --- | --- | --- | --- | --- | --- |
| aPM1 | 179.0714_9.91 | **<0.001** | 95.8% | 17385 | 1.8% |
| aPM2a | 327.1449_12.50 | **<0.001** | 75.0% | 13299 | 0.3% |
| aPM2b | 327.1449_12.65 | **<0.001** | 100.0% | 461614 | 14.6% |
| aPM2c | 327.1449_12.83 | **<0.001** | 95.8% | 154854 | 4.9% |
| aPM3a | 341.1242_7.37 | **<0.001** | 93.8% | 50629 | 3.2% |
| aPM3b | 341.1242_12.59 | **<0.001** | 100.0% | 352366 | 18.2% |
| aPM4a | 343.1398_7.98 | **<0.001** | 89.6% | 72991 | 2.4% |
| aPM4b | 343.1398_8.85 | **<0.001** | 81.3% | 14109 | 1.2% |
| aPM4c | 343.1398_10.99 | **<0.001** | 89.6% | 5156 | 0.5% |
| aPM4d | 343.1398_12.78 | **<0.001** | 97.9% | 183468 | 15.4% |
| aPM4e | 343.1398_12.85 | **<0.001** | 95.8% | 241782 | 25.2% |
| aPM5a | 357.1191_5.05 | **<0.001** | 79.2% | 4787 | 0.3% |
| aPM5b | 357.1191_6.47 | **<0.001** | 91.7% | 22121 | 1.6% |
| aPM5c | 357.1191_6.53 | **<0.001** | 79.2% | 25963 | 1.2% |
| aPM5d | 357.1191_6.83 | **<0.001** | 81.3% | 5115 | 0.4% |
| aPM5e | 357.1191_7.63 | **<0.001** | 72.9% | 3744 | 0.3% |
| aPM5f | 357.1191_7.79 | **<0.001** | 87.5% | 5543 | 0.5% |
| aPM5g | 357.1191_8.14 | **<0.001** | 100.0% | 77327 | 4.4% |
| aPM6 | 359.1348_7.41 | **<0.001** | 91.7% | 17445 | 1.1% |
| aPM7a | 361.1504_4.85 | **<0.001** | 89.6% | 20611 | 1.3% |
| aPM7b | 361.1504_5.27 | **<0.001** | 83.3% | 8663 | 0.2% |
| aPM8 | 373.1140_3.10 | **<0.001** | 83.3% | 13644 | 0.8% |

* ANOVA result, bold if p<0.05

Frequency of occurrence: percentage of samples with the specific analyte detected. For each LM, “Frequency of occurrence (%)” was calculated based on the sample number after exposure (40 for α-pinene inhalation). “Maximum response” was highest peak response across all urine samples. “Proportion (observed)” were calculated by three steps (“j” was an index for a urine sample; “x” for was an index for a particular LM):

- Adding responses of all 22 aPM from a sample to get the “Total aPM response” of this specific sample:

$${Total\_aPM\_Response}_{i}= \sum_{x=1}^{22} {aPM\_Response}_{ix}$$

- Dividing response of one particular LM by the “Total aPM response” of a sample to get the “Proportion” for this LM in that specific sample:

$$\mathrm{Proportion}_{ix}= \frac{{aPM\_Response}_{ix}}{{Total\_aPM\_Response}_{i}}$$

- Averaging all “Proportion” from all samples (after exposure) to get the “Proportion (observed)”:

$$\mathrm{Proportion}_{\mathrm{observed}}= \frac{\sum_{i=1}^{n} \mathrm{Proportion}_{ix}}{n}$$

**Table S6.** aPM detected in mouse urine collected after (+) α-pinene or (-) α-pinene i.p. injection

| ID | (1R)-(+)-α-Pinene  (Average) | (1S)-(−)-α-Pinene  (Average) | R/(R+S) * | Assigned peak identity |
| --- | --- | --- | --- | --- |
| aPM1 | 0.1% | 0.8% | 12.9% | MYRAO |
| aPM2a | 0.0% | 0.0% | n/a | aPIN-O-GlcA_Rt12.50 |
| aPM2b | 50.5% | 34.4% | 59.5% | aPIN-O-GlcA_Rt12.65 |
| aPM2c | 21.8% | 30.3% | 41.8% | aPIN-O-GlcA_Rt12.83 |
| aPM3a | 0.0% | 10.0% | 0.0% | MYRA-GlcA_Rt7.37 |
| aPM3b | 1.6% | 2.7% | 37.5% | MYRA-GlcA_Rt12.59 |
| aPM4a | 0.0% | 5.0% | 0.0% | DHMYRA-GlcA_Rt7.98 |
| aPM4b | 0.0% | 4.2% | 0.0% | DHMYRA-GlcA_Rt8.85 |
| aPM4c | 0.0% | 0.1% | 0.0% | DHMYRA-GlcA_Rt10.99 |
| aPM4d | 0.0% | 0.2% | 0.0% | DHMYRA-GlcA_Rt12.78 |
| aPM4e | 0.1% | 0.2% | 24.1% | DHMYRA-GlcA_Rt12.85 |
| aPM5a | 0.0% | 0.1% | 0.0% | MYRA-4-O-GlcA_Rt5.05 |
| aPM5b | 0.0% | 0.0% | n/a | MYRA-4-O-GlcA_Rt6.47 |
| aPM5c | 0.0% | 0.0% | n/a | MYRA-4-O-GlcA_Rt6.53 |
| aPM5d | 0.2% | 0.0% | 100.0% | MYRA-4-O-GlcA_Rt6.83 |
| aPM5e | 0.0% | 0.3% | 0.0% | MYRA-4-O-GlcA_Rt7.63 |
| aPM5f | 0.0% | 0.0% | n/a | MYRA-4-O-GlcA_Rt7.79 |
| aPM5g | 0.0% | 0.0% | n/a | MYRA-4-O-GlcA_Rt8.14 |
| aPM6 | 0.8% | 8.0% | 9.4% | DHMYRA-4-O-GlcA |
| aPM7a | 21.2% | 3.8% | 84.7% | PNRL-GlcA_Rt4.85 |
| aPM7b | 3.7% | 0.0% | 100.0% | PNRL-GlcA_Rt5.27 |
| aPM8 | 0.0% | 0.0% | n/a | MYRA-4-O-7-O-GlcA |

*: “n/a” indicates metabolites that were undetected in mice following exposure to either (+) α-pinene or (-) α-pinene.

**Table S7**. Response of aPM in human urine after greenness exposure

| ID | p-value* | Frequency of occurrence | Maximum response | Proportion (observed) after greenness exposure |
| --- | --- | --- | --- | --- |
| aPM1 | 0.409 | 31.3% | 3544 | 1.3% |
| aPM2a | 0.248 | 25.0% | 445 | 0.9% |
| aPM2b | n/a | 6.3% | 171 | 0.3% |
| aPM2c | 0.994 | 31.3% | 1435 | 0.5% |
| aPM3a | n/a | 0.0% | n/a | n/a |
| aPM3b | **0.037** | 87.5% | 2516 | 17.7% |
| aPM4a | n/a | 6.3% | 53 | 0.0% |
| aPM4b | n/a | 0.0% | n/a | n/a |
| aPM4c | n/a | 0.0% | n/a | n/a |
| aPM4d | 0.075 | 68.8% | 7375 | 9.4% |
| aPM4e | **0.022** | 93.8% | 27114 | 67.8% |
| aPM5a | n/a | 0.0% | n/a | n/a |
| aPM5b | n/a | 6.3% | 89 | 0.0% |
| aPM5c | 0.117 | 37.5% | 136 | 0.7% |
| aPM5d | n/a | 0.0% | n/a | n/a |
| aPM5e | 0.057 | 25.0% | 93 | 1.0% |
| aPM5f | n/a | 0.0% | n/a | n/a |
| aPM5g | **0.043** | 25.0% | 98 | 0.4% |
| aPM6 | n/a | 6.3% | 178 | 0.0% |
| aPM7a | n/a | 0.0% | n/a | n/a |
| aPM7b | n/a | 0.0% | n/a | n/a |
| aPM8 | n/a | 0.0% | n/a | n/a |

*: t-Test result; bold if p < 0.05; n/a for aPMs not detected, or detected in less than 20% of the samples

Frequency of occurrence: percentage of samples with the specific analyte detected. For each aPM, “Frequency of occurrence (%)” was calculated based on the sample number after exposure (8 for greenness exposure). “Maximum response” and “Proportion (observed)” were calculated using the method same as Tables S4

**Table S8**. P-values of t-test after removal of one or two IDs with the highest background of aPM

| Figure | Metabolite ID | Metabolites | No removal | Remove one ID with the highest background | Remove two IDs with the highest background |
| --- | --- | --- | --- | --- | --- |
| 4A | aPM2b | aPIN.O.GlcA_Rt12.65 | 0.187 | 0.246 | 0.473 |
| 4B | aPM3b | MYRA.GlcA_12.59 | 0.037 | 0.016 | 0.005 |
| 4C | aPM4d | DHMYRA.GlcA_12.78 | 0.075 | 0.029 | 0.028 |
| 4D | aPM4e | DHMYRA.GlcA_12.85 | 0.022 | 0.007 | 0.006 |

***Supplementary Figures***

***
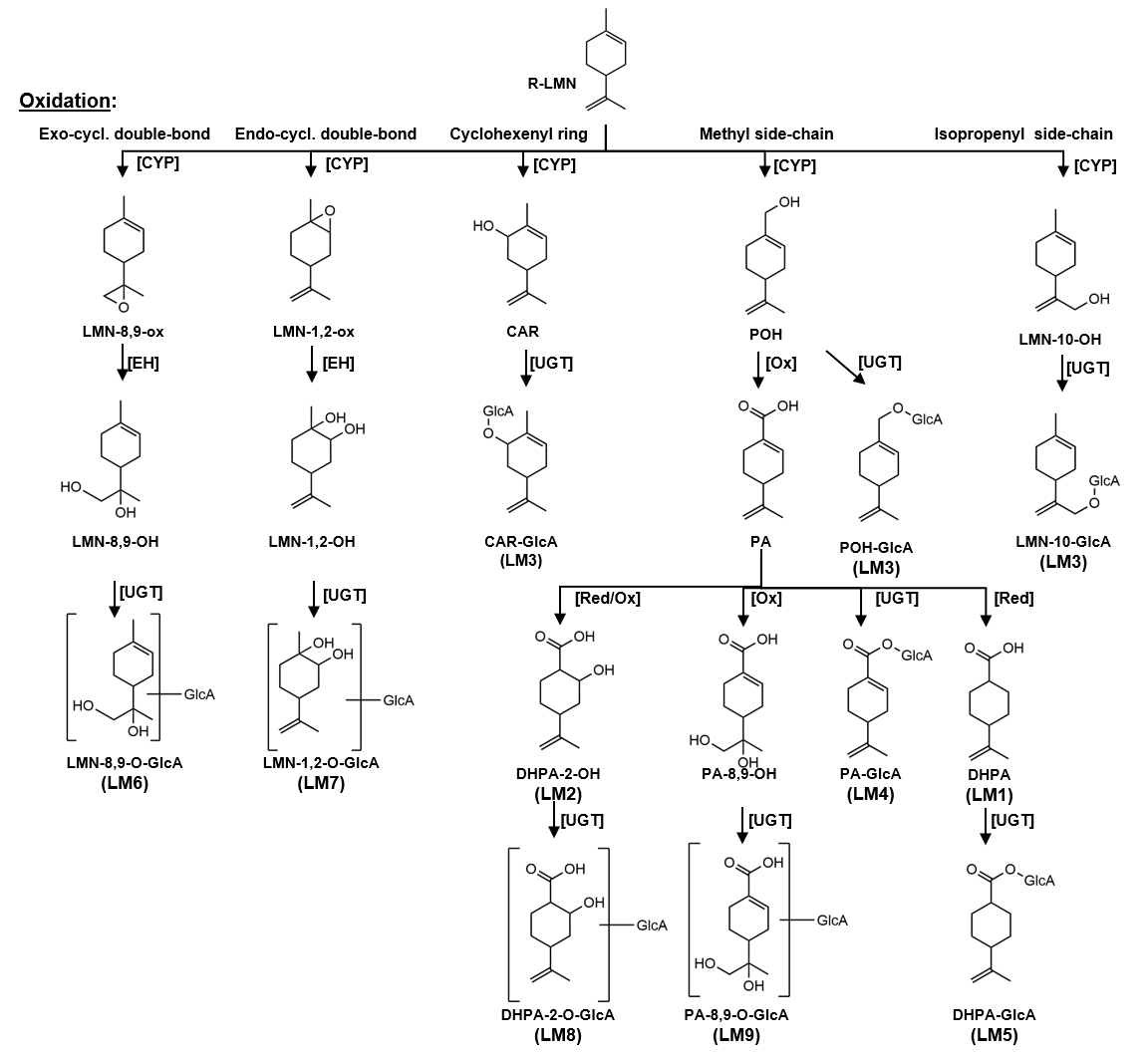
***

**Figure S1.** Limonene metabolism pathway in human (Adopted from [2] with modifications) . Only detected metabolites (with LM numbers) and their intermediate products are shown in the figure. Abbreviations: limonene (LMN); glucuronic acid (GlcA); glycine (Gly); epoxide hydrolase (EH); oxidation (Ox); reduction (Red); UDP-glucuronosyl transferase (UGT); cytochromes P450 (CYP).


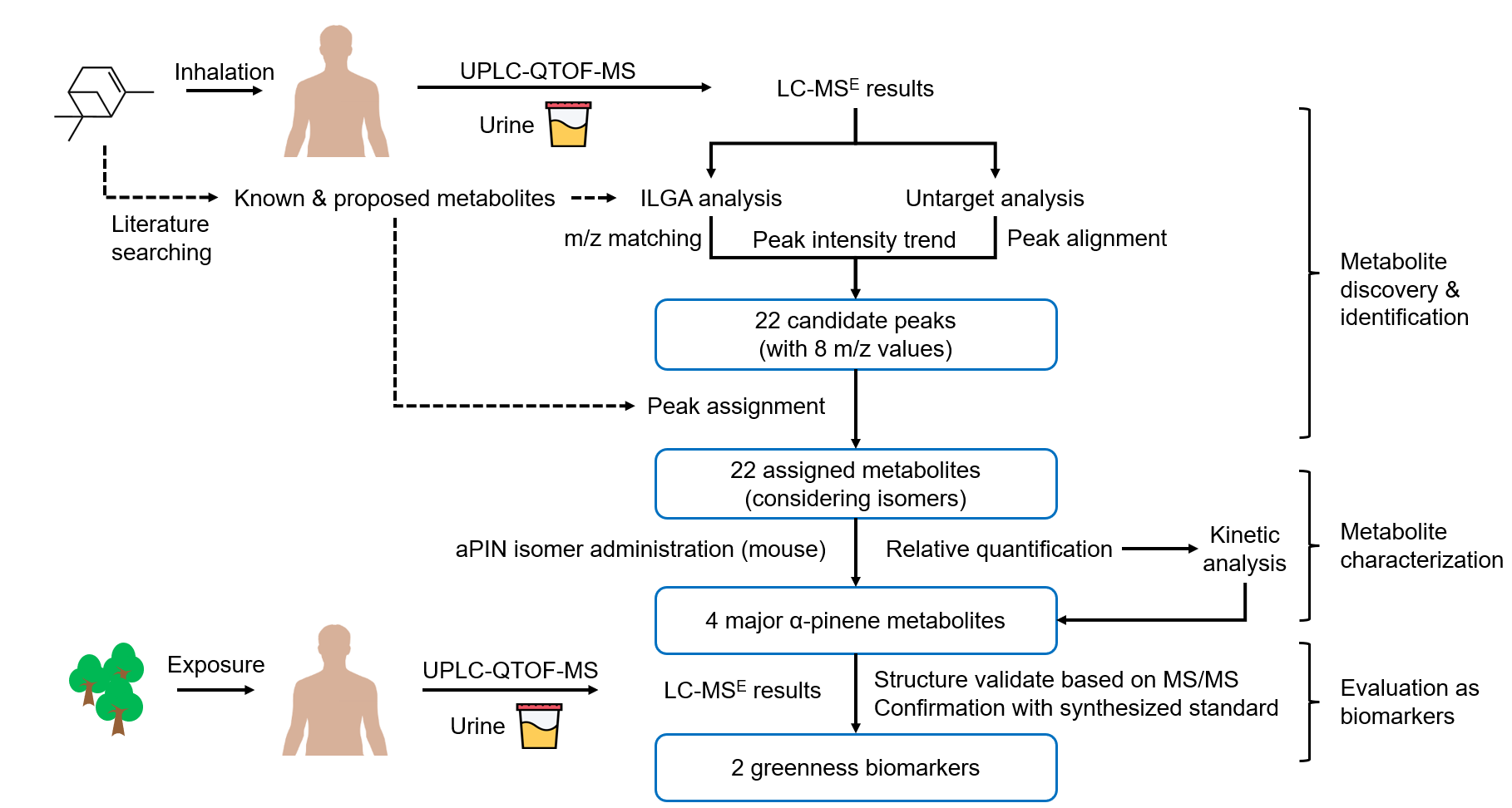


**Figure S2.** Workflow for evaluation of α-pinene metabolites (aPM) as biomarkers of exposure to greenness

**
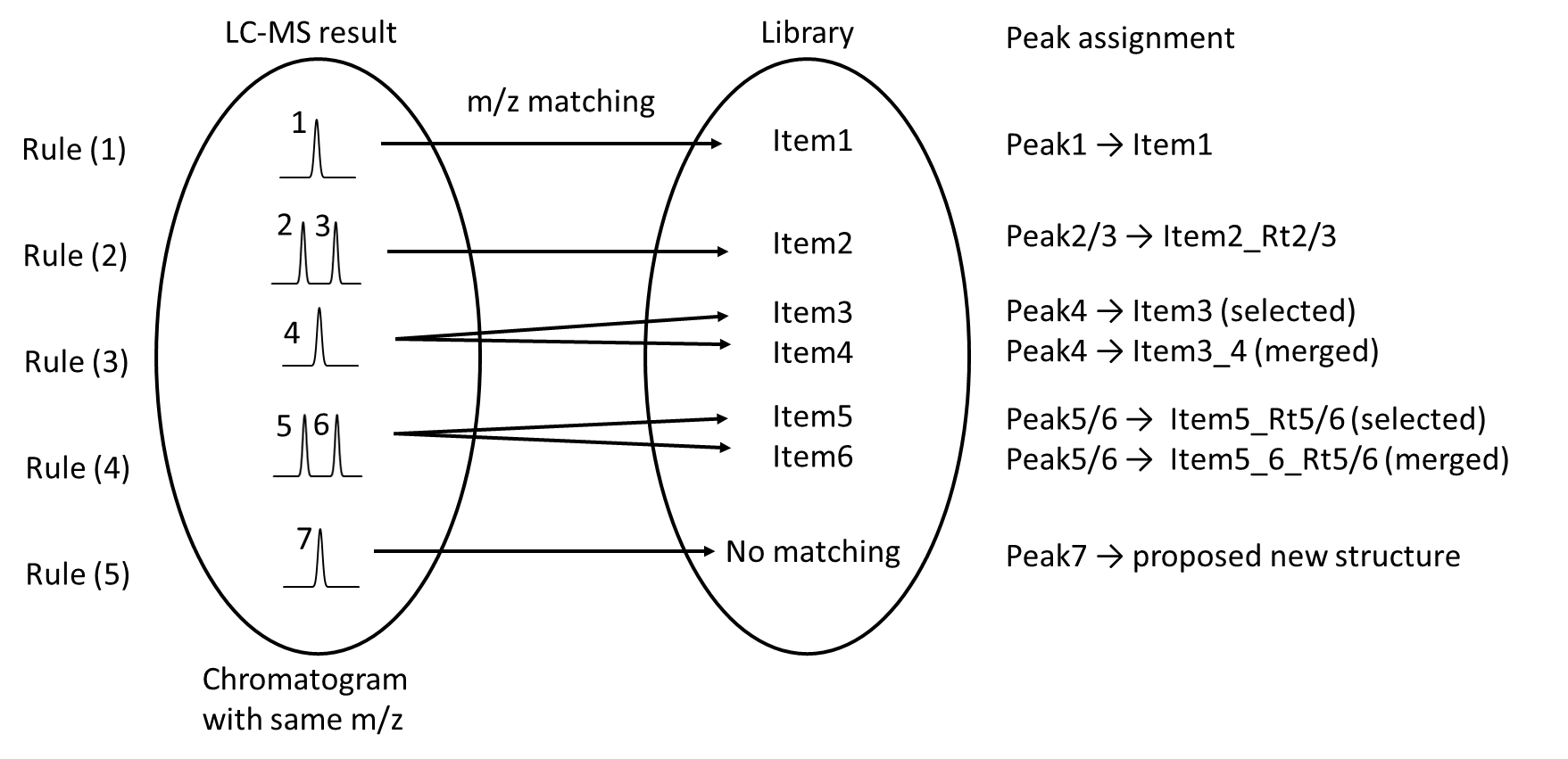
**

**Figure S3.** Rules for peak assignment. Five rules were designed to address the isomer issue for peak assignment. According to these rules, library items were allocated to LC-MS peaks with matching m/z values. The five rules are:

1. **One peak to one library item**: A peak with one matching item in the scouting library was assigned as is. (This rule is not changed.)
2. **Many peaks (with same m/z) to one library item**: For multiple peaks with one matching item, each peak represented an isomer that is indistinguishable in the library. To differentiate these isomers in the assignment, annotation for each peak was supplemented with its respective retention time (Rt) value. (This rule is not changed.)
3. **One peak to many library items (with same MW)**: For a peak with two or more matching entries, the item derived from previously reported human urinary phase I metabolite was chosen (3a: selection). If such a selection was not possible, these entries were merged into one label (3b: combination). (This rule merged previous rule 3 and 4)
4. **Many peaks to many m/z**: For multiple peaks with two or more matching entries, rule 3 was applied for identity selection or combination, then rule 2 was applied for peak assignment. (This rule clarifies the application of rules 2 and 3 in this scenario.)
5. **Peaks with no matching m/z in the library**: For a peak found by untargeted analysis but having no matching m/z in the library, a new structure was proposed based on its mass shifts related to identified metabolites displaying strong correlation in levels. (This is a new rule to cover untargeted analysis findings.)

**Figure S4.** Fitting NLME models to urinary levels of metabolite aPM3b after α-pinene inhalation. This figure compares the input values (dots) and predicted values (polylines) based on the fitting result of NLME model for metabolite aPM3b in each individual precipitant (subject BV100 to 107).


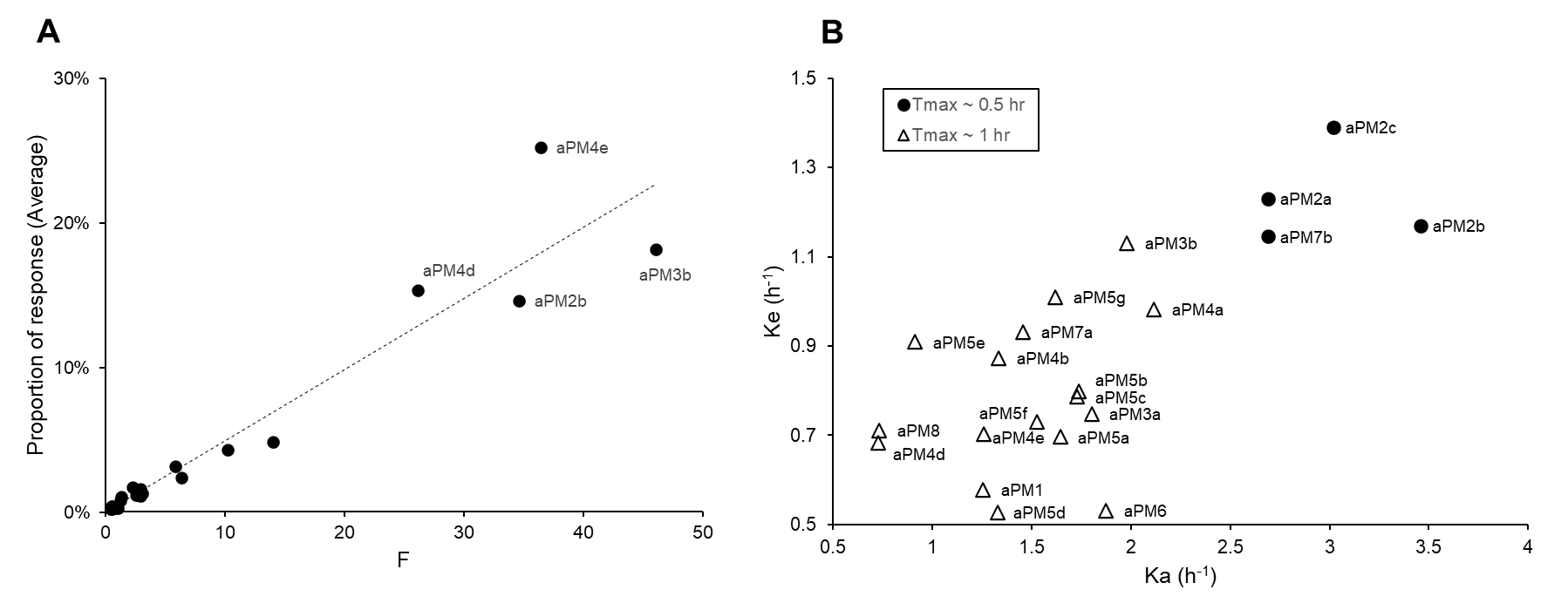


**Figure S5**. Comparison of relative quantification and kinetic analysis results. **A.** Comparing the F value vs. Proportion (observed) for each aPM. The 4 major aPM are labeled with their IDs and identities. **B**. Comparing Ka and Ke values with peak time (T_max_) of each aPM. All aPM are labeled with their IDs.


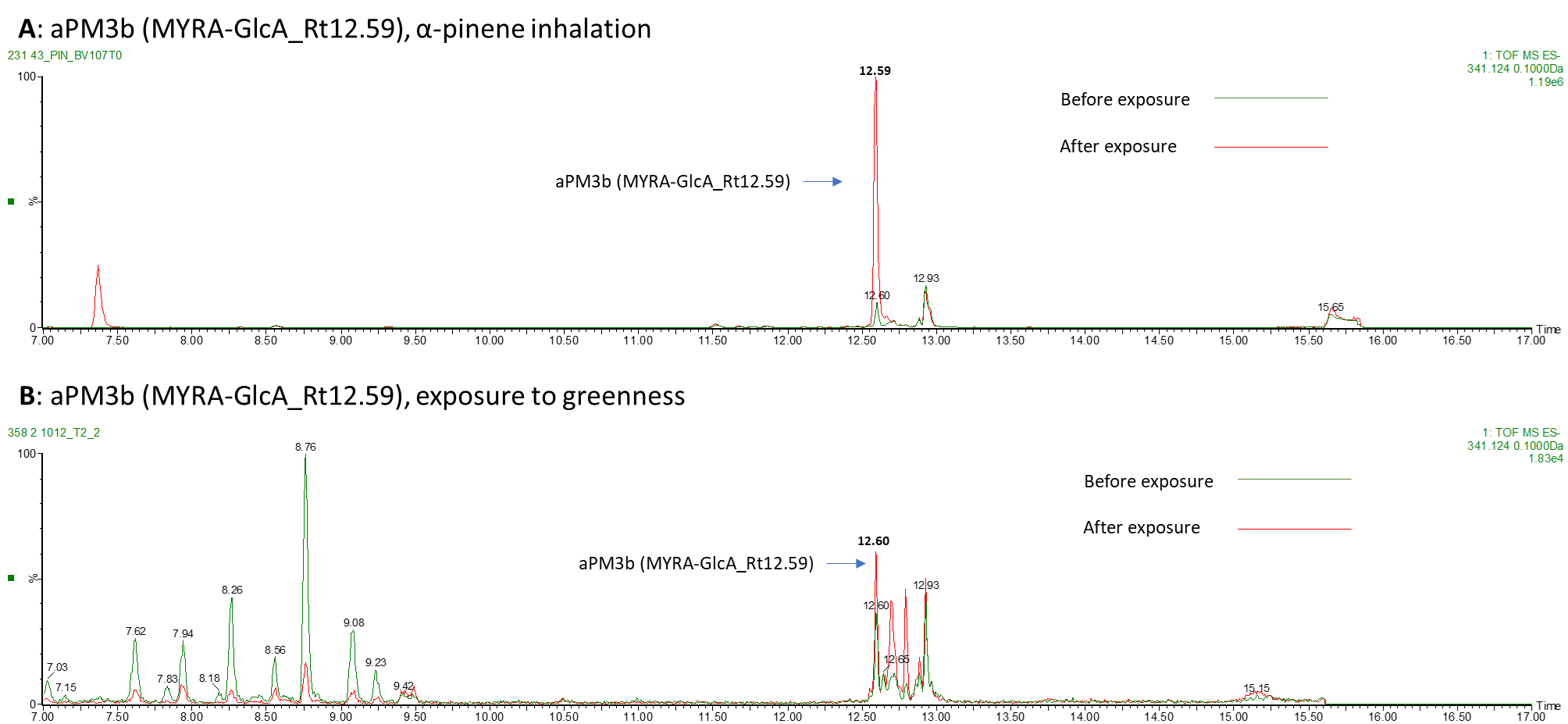


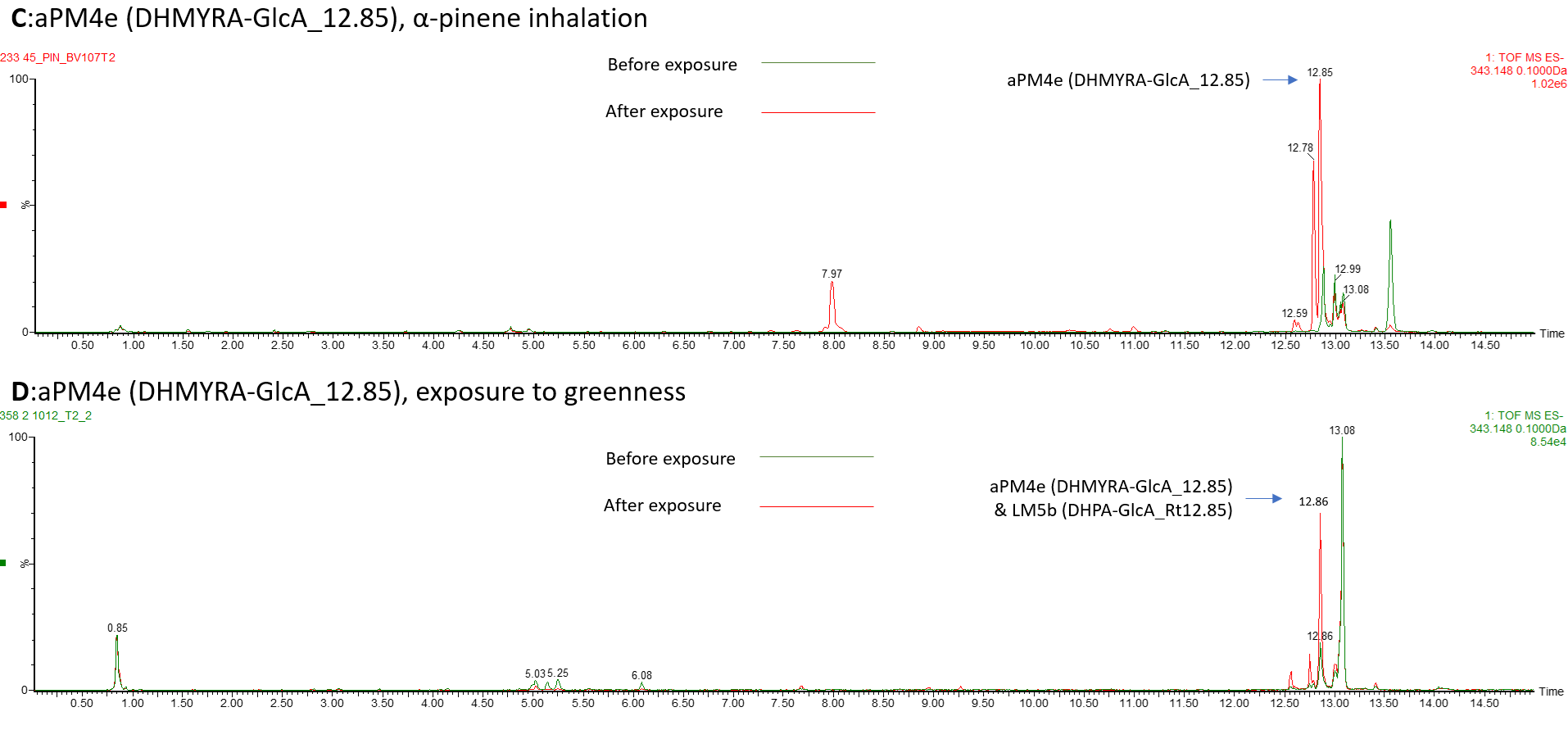


**Figure S6.** Chromatography of aPM3b (MYRA-GlcA_Rt12.59, myrtenic acid-GlcA) and aPM4e (DHMYRA-GlcA_12.85, dihydromyrtenic acid-GlcA) in human urine before or after exposure to α-pinene or greenness. **A**. aPM3b (MYRA-GlcA_Rt12.59, myrtenic acid-GlcA) in urine after α-pinene inhalation; **B**. aPM3b (MYRA-GlcA_Rt12.59, myrtenic acid-GlcA) in urine after exposure to greenness; **C**. aPM4e (DHMYRA-GlcA_12.85, dihydromyrtenic acid-GlcA) in urine after α-pinene inhalation; **D**. aPM4e (DHMYRA-GlcA_12.85, dihydromyrtenic acid-GlcA) in urine after exposure to greenness. Please notice that aPM4e (DHMYRA-GlcA_12.85, dihydromyrtenic acid-GlcA) and limonene metabolite LM5b (DHPA-GlcA_Rt12.85, dihydroperillic acid glucuronide) have the same retention time and cannot distinguish under current LC-MS conditions. Green color: before exposure; red color: after exposure.


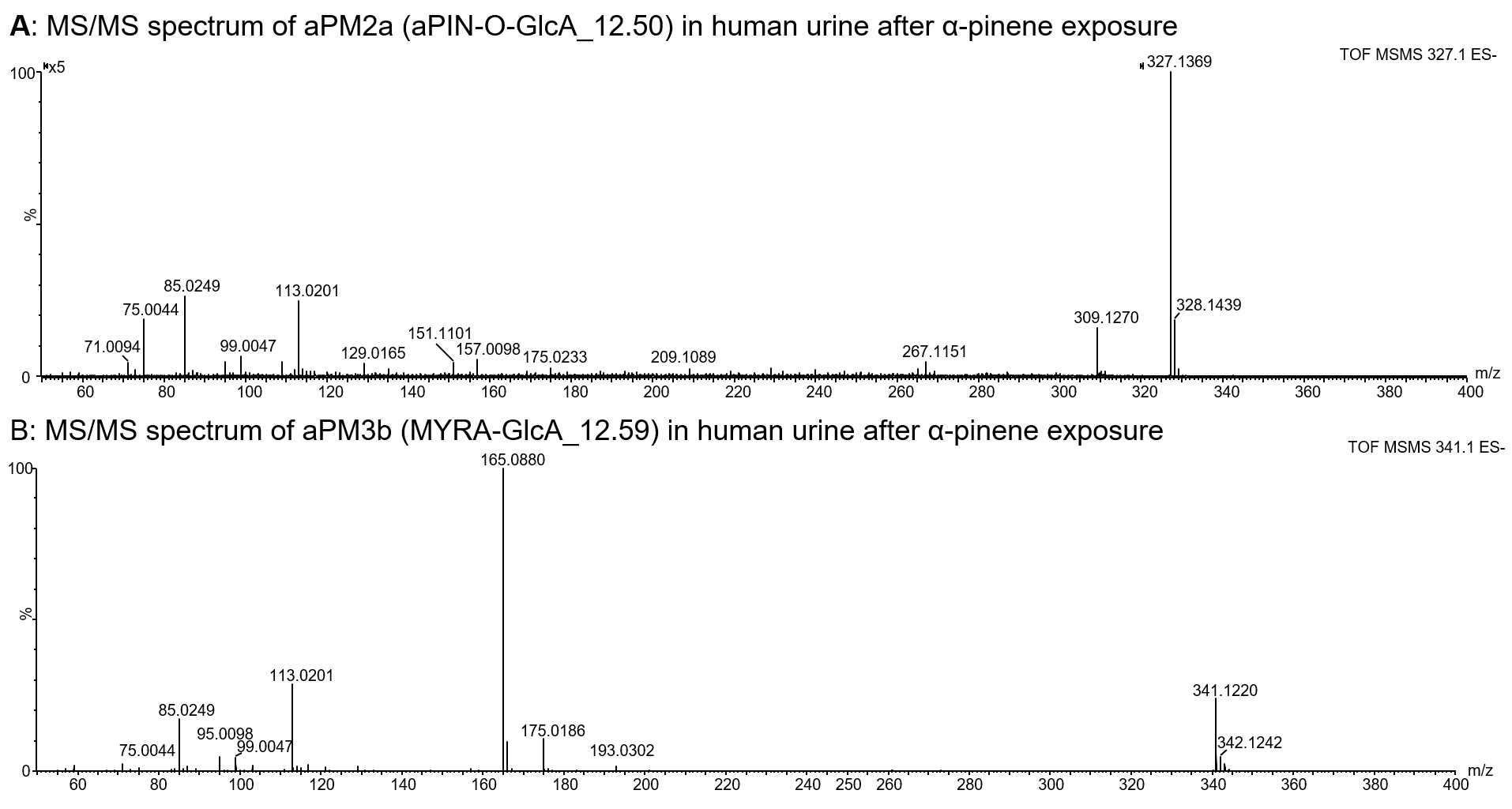


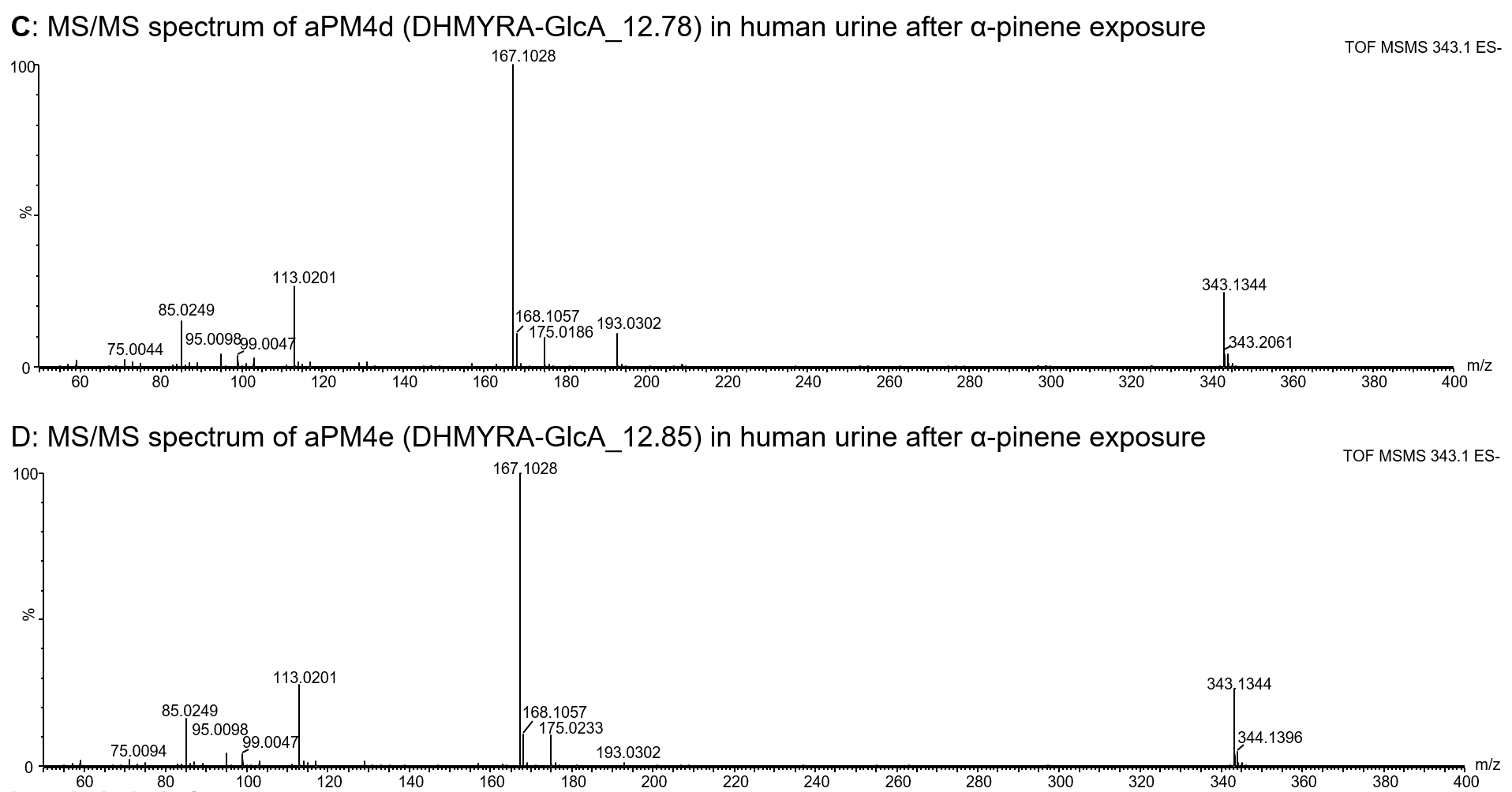


**Figure S7.** MS/MS spectra of some aPMs in human urine. **A**. aPM2a (aPIN-O-GlcA_12.50, α-pinene-O-GlcA). The signal intensity was magnified 5 times over the range of 50-320 m/z; **B**. aPM3b (MYRA-GlcA_Rt12.59, myrtenic acid-GlcA); **C**. aPM4 (DHMYRA-GlcA_12.78, dihydromyrtenic acid-GlcA); **D**. aPM4e (DHMYRA-GlcA_12.85, dihydromyrtenic acid-GlcA)


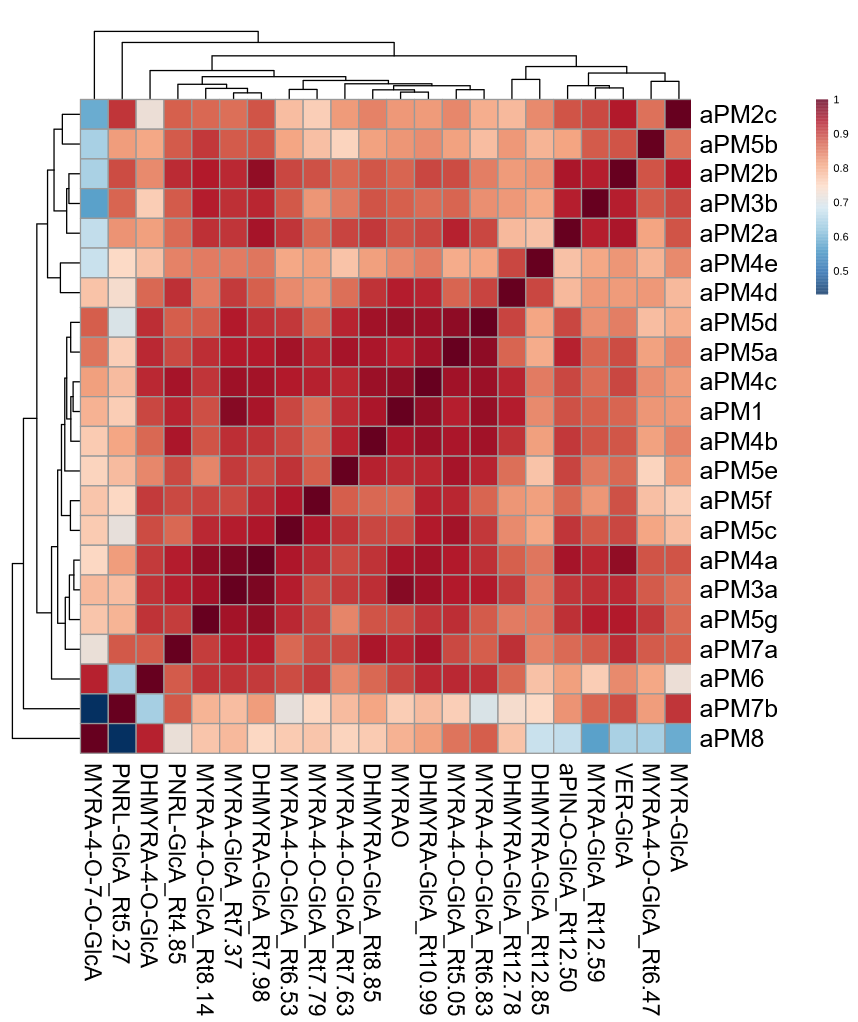


**Figure S8.** Correlation heatmaps of 22 aPMs discovery in human urine. The row names are Metabolite IDs and column names are Assigned peak identity (**Table 1**). This heatmap was generated using MetaboAnalyst 6.0. It depicts the Pearson correlation coefficients (r) between various aPMs based on their normalized responses to urinary creatinine for each subject. After normalization, the data underwent log transformation to enhance the interpretability and reduce skewness. In the heatmap, the color scheme indicates the strength and direction of the correlations: higher r values (indicating stronger positive correlations) are represented in red, while lower r values (indicating weaker or negative correlations) are shown in blue. The full names of abbreviations: MYRAO: Myrtenic acid-4-one; aPIN-O-GlcA: α-Pinene-O-GlcA; VER-GlcA: Myrtenol-GlcA; VER-GlcA: Verbenol-GlcA; MYRA-GlcA: Myrtenic acid-GlcA; DHMYRA-GlcA: Dihydromyrtenic acid-GlcA; MYRA-4-O-GlcA: Myrtenic acid-4-O-GlcA; DHMYRA-4-O-GlcA: Dihydromyrtenic acid-4-O-GlcA; PNRL-GlcA: Pinanetriol-GlcA; MYRA-4-O-7-O-GlcA: Myrtenic acid-4-O-7-O-GlcA

***References***

1. Xie, Z., et al., *Global Profiling of Urinary Mercapturic Acids Using Integrated Library-Guided Analysis.* Environ Sci Technol, 2023. **57**(29): p. 10563-10573.

2. Xie, Z., et al., *Evaluation of urinary limonene metabolites as biomarkers of exposure to greenness.* Environmental Research, 2024. **245**: p. 117991.

3. Chen, C.J., et al., *Recent advances in LC-MS-based metabolomics for clinical biomarker discovery.* Mass Spectrom Rev, 2023. **42**(6): p. 2349-2378.

4. Benito, S., et al., *Untargeted metabolomics for plasma biomarker discovery for early chronic kidney disease diagnosis in pediatric patients using LC-QTOF-MS.* Analyst, 2018. **143**(18): p. 4448-4458.

5. Cui, L., H. Lu, and Y.H. Lee, *Challenges and emergent solutions for LC-MS/MS based untargeted metabolomics in diseases.* Mass Spectrom Rev, 2018. **37**(6): p. 772-792.

6. Zhang, J., et al., *An intelligentized strategy for endogenous small molecules characterization and quality evaluation of earthworm from two geographic origins by ultra-high performance HILIC/QTOF MS E and Progenesis QI.* Analytical and bioanalytical chemistry, 2016. **408**: p. 3881-3890.

7. Liao, J., et al., *Different software processing affects the peak picking and metabolic pathway recognition of metabolomics data.* J Chromatogr A, 2023. **1687**: p. 463700.

8. Schmidt, L. and T. Goen, *Human metabolism of alpha-pinene and metabolite kinetics after oral administration.* Arch Toxicol, 2017. **91**(2): p. 677-687.

9. Evich, M.G., et al., *Untargeted MS(n)-Based Monitoring of Glucuronides in Fish: Screening Complex Mixtures for Contaminants with Biological Relevance.* ACS ES T Water, 2022. **2**(12): p. 2481-2490.

10. Rinaldi de Alvarenga, J.F., et al., *Identification of D-Limonene Metabolites by LC-HRMS: An Exploratory Metabolic Switching Approach in a Mouse Model of Diet-Induced Obesity.* Metabolites, 2022. **12**(12).

11. Basu, S., et al., *Sparse network modeling and metscape-based visualization methods for the analysis of large-scale metabolomics data.* Bioinformatics, 2017. **33**(10): p. 1545-1553.

12. Cai, Y.P., Z.W. Zhou, and Z.J. Zhu, *Advanced analytical and informatic strategies for metabolite annotation in untargeted metabolomics.* Trac-Trends in Analytical Chemistry, 2023. **158**.

13. Li, X., et al., *Two New Monoterpenes from Tithonia diversifolia and Their Anti-Hyperglycemic Activity.* Records of Natural Products, 2013. **7**(4): p. 351-354.

14. Scapolla, C., et al., *Identification and structural characterization by LC-ESI-IONTRAP and LC-ESI-TOF of some metabolic conjugation products of homovanillic acid in urine of neuroblastoma patients.* J Mass Spectrom, 2012. **47**(7): p. 816-24.

15. Han, Z., et al., *In vitro glucuronidation of ochratoxin a by rat liver microsomes.* Toxins (Basel), 2013. **5**(12): p. 2671-85.

16. Waidyanatha, S., et al., *The common indoor air pollutant alpha-pinene is metabolised to a genotoxic metabolite alpha-pinene oxide.* Xenobiotica, 2022. **52**(3): p. 301-311.

17. Eriksson, K. and J.O. Levin, *Gas chromatographic-mass spectrometric identification of metabolites from alpha-pinene in human urine after occupational exposure to sawing fumes.* J Chromatogr B Biomed Appl, 1996. **677**(1): p. 85-98.

18. Schmidt, L., V.N. Belov, and T. Göen, *Sensitive monitoring of monoterpene metabolites in human urine using two-step derivatisation and positive chemical ionisation-tandem mass spectrometry.* Anal Chim Acta, 2013. **793**: p. 26-36.

19. Ishida, T., et al., *Terpenoids biotransformation in mammals III: Biotransformation of alpha-pinene, beta-pinene, pinane, 3-carene, carane, myrcene, and p-cymene in rabbits.* J Pharm Sci, 1981. **70**(4): p. 406-15.

20. Southwell, I.A., T.M. Flynn, and R. Degabriele, *Metabolism of alpha- and beta-pinene, p-cymene and 1,8-cineole in the brushtail possum, Trichosurus vulpecula.* Xenobiotica, 1980. **10**(1): p. 17-23.

21. Yang, H.D., et al., *Monoterpenoids from the root bark of Acanthopanax gracilistylus and their inhibitory effects on neutrophil elastase, 5-lipoxygenase, andcyclooxygenase-2 in vitro.* Phytochemistry, 2023. **215**: p. 113851.
